# Modularity and stability in ecological communities

**DOI:** 10.1038/ncomms12031

**Published:** 2016-06-23

**Authors:** Jacopo Grilli, Tim Rogers, Stefano Allesina

**Affiliations:** 1 Department of Ecology & Evolution, University of Chicago. 1101 E. 57th, Chicago, Illinois 60637, USA; 2 Centre for Networks and Collective Behaviour, Department of Mathematical Sciences, University of Bath. Claverton Down Bath BA2 7AY United Kingdom; 3 Computation Institute, University of Chicago, Chicago, Illinois 60637, USA; 4 Northwestern Institute on Complex Systems (NICO), Northwestern University, Evanston, Illinois 60208, USA

## Abstract

Networks composed of distinct, densely connected subsystems are called modular. In ecology, it has been posited that a modular organization of species interactions would benefit the dynamical stability of communities, even though evidence supporting this hypothesis is mixed. Here we study the effect of modularity on the local stability of ecological dynamical systems, by presenting new results in random matrix theory, which are obtained using a quaternionic parameterization of the cavity method. Results show that modularity can have moderate stabilizing effects for particular parameter choices, while anti-modularity can greatly destabilize ecological networks.

## Abstract (web summary)

Modularity in food webs can be caused by spatial and temporal mismatches in interactions. Here, Jacopo Grilli, Tim Rogers and Stefano Allesina show that modularity, contrary to expectations, does not generally help stabilizing ecological communities.

## Graphical abstract

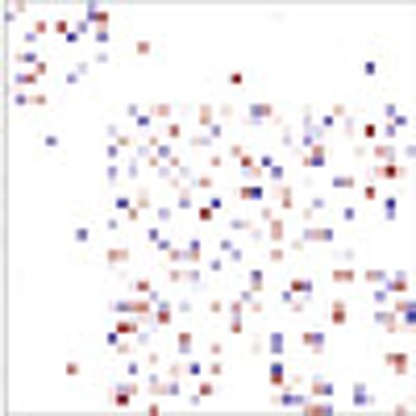

Ecological communities are structured both in space, as in a stratified lake and in time, as for migratory birds. This temporal and spatial organization is reflected in the strength of species interactions, where we expect populations dwelling in the same location, or being active in the same season, to interact more frequently than those that are not.

When drawing ecological interaction networks—where species are the nodes and the edges connecting the species stand for interactions (for example, consumption, pollination and competition)—we therefore expect to find that species can be partitioned into distinct ‘groups', such that the frequency of interaction largely depends on group-membership^1^,^2^. Networks with this property are said to be ‘block-structured': a modular structure is a particular block structure, in which a network is divided into subsystems, and within-subsystem interactions are much more frequent than those between subsystems^3^,^4^. If, on the other hand, interactions occur exclusively between groups, we obtain a bipartite network—another type of block structure with many ecological applications^5^.

The appealingly simple idea of a block structure has been formalized in the measure of modularity, *Q*^3^,^4^, calculated as the difference between observed and expected within-group interactions, divided by the total number of interactions. Positive values indicate that interactions occur predominantly within-groups, while negative values that interactions are more frequent between- than within-group. Modularity has become one of the most investigated network metrics, with applications spanning biological, social and technological networks^6^,^7^,^8^,^9^,^10^,^11^.

Unveiling the relationship between the network structure of biological systems and their dynamical properties has been a long-sought goal of the discipline, and many authors have hypothesized that biological networks must be shaped by evolution^12^,^13^,^14^,^15^ and co-evolution^16^, favouring configurations yielding controlled dynamics^17^,^18^,^19^.

In ecology, the idea that a modular organization would be beneficial for the local stability of ecological communities (that is, their ability to recover from small perturbations) dates back to work on complexity and stability by May^20^ , where he suggested ‘that our model multi-species communities [...] will do better if the interactions tend to be arranged in blocks'. This hypothesis was challenged by a number of authors^21^,^22^, who produced simulations showing the opposite effect. However, recent studies found that modularity can indeed enhance species persistence^23^,^24^, further complicating the picture. Though the relationship between block structure and dynamics on networks has since been investigated in many fields, including epidemiology^25^, neuroscience^26^ and complex systems in general^27^, a systematic classification is still lacking.

Here we study how the local stability of ecological systems is influenced by modularity, providing new results on the theory of random matrices that can be used to draw a direct relationship between modularity *Q* and local stability. The mathematical results are briefly stated in the Methods section, while a calculation based on the cavity method^28^,^29^,^30^,^31^ with quaternions is carried out in the Supplementary Notes.

We show that, with respect to the corresponding unstructured case, modularity can have moderate stabilizing effects for particular parameter choices, while anti-modularity can greatly destabilize networks. The rich range of possible effects associated with the same level of modularity stresses the fact that a given network structure is not ‘stabilizing' or ‘destabilizing' *per se*, but only for particular regimes.

## Results

### Building community matrices

We study the stability of a community matrix *M*, modelling a continuous-time, dynamical ecological system composed of *S* populations, resting at a feasible equilibrium point. We remove self-interactions from the matrix (setting *M*_*ii*_=0), so that we can concentrate on inter-specific effects (adding intra-specific effects would not qualitatively alter the results^32^).

**Figure 1:**
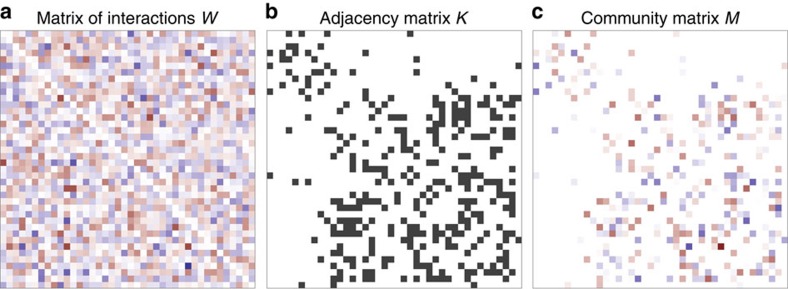
Block structure. (**a**) The matrix of interaction strengths *W*, whose diagonal elements are all zero and the off-diagonal elements are independently sampled in pairs from a bivariate distribution (in this case, a bivariate normal with identical marginals defined by *μ*=0, *σ*=1/2 and correlation *ρ*=−3/4; red stands for negative coefficients, blue for positive one and the intensity of the colour is proportional to the coefficient value). (**b**) The adjacency matrix *K*, which has a block structure; in this case, we have that *α*=1/4 of the species belong to the first subsystem, and that the modularity *Q*=0.3, meaning that interactions tend to occur within-subsystem. (**c**) The community matrix *M* is obtained by multiplying *W* and *K* element-by-element.

*M* is obtained by multiplying element-by-element two matrices, a matrix of interaction strengths, *W*, where *W*_*ij*_ expresses the effect of species *j* on species *i* around equilibrium, and the adjacency matrix of an undirected graph, *K*. The community matrix is therefore *M*=*W* ○ *K* (Fig. 1).

Initially, we independently sample the coefficients in *W* in pairs, drawing (*W*_*ij*_, *W*_*ji*_) from a bivariate distribution with identical marginals, defined by the mean 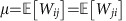, the variance 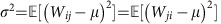 and the correlation 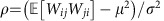. By varying these parameters, we can model different types of interactions between the species from preponderantly predator–prey to dominated by competition or mutualism^33^.

The binary matrix *K* dictates ‘who interacts with whom', and is symmetric because we assume pairwise interactions. Thus, *K* determines which interactions in *W* are activated, and which are suppressed. Here we study the case of a block-structured *K*: we assume that the community is composed of two subsystems, of sizes *αS* and (1−*α*)*S*, respectively (with *α*≤1/2), and that species in the same subsystem interact with probability *C*_w_ (within-subsystem connectance), while species in different subsystems with probability *C*_b_ (between-subsystem connectance).

**Figure 2:**
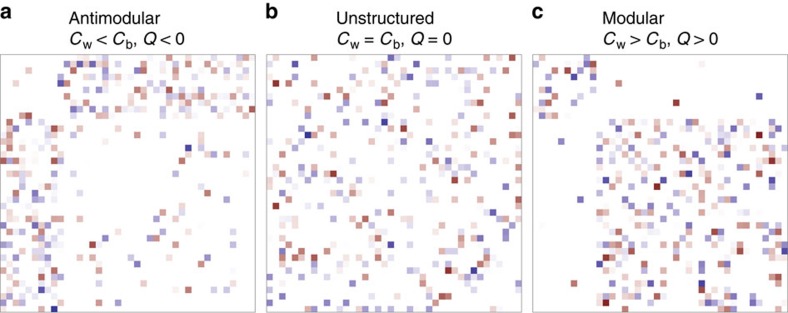
Modularity. By varying the modularity *Q* (or, equivalenty, the within-group connectance *C*_w_ and between-group connectance *C*_b_), we can produce community matrices where interactions occur mostly between species in different subsystems (*Q*<0, **a**), completely at random (*Q*=0, **b**) or mostly within subsystems (*Q*>0, **c**).

This parameterization is especially intuitive, because for *C*_b_=*C*_w_, we recover the well-studied case of a random ecological community^20^,^33^. Hence, by varying *C*_b_ and *C*_w_, we can isolate the effect of having a modular or anti-modular structure (Fig. 2), with the two extreme cases being networks composed of two separate subsystems (perfectly modular), and those in which interactions occur exclusively between subsystems (perfectly anti-modular or bipartite). For simplicity, we speak of a ‘modular' structure whenever *C*_w_>*C*_b_, and of an ‘anti-modular' structure when *C*_w_<*C*_b_. The case of *C*_w_=*C*_b_ represents ‘unstructured' systems, such as those studied by May and other authors^20^,^33^. Equivalently (Methods), we can express *C*_w_ and *C*_b_ in terms of the overall connectance *C* (that is, the overall density of interactions in *K*) and *Q*, the modularity of the network^3^,^4^, defined as:


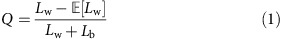


where *L*_w_ is the observed number of interactions within the subsystems, *L*_b_ the number of between-subsystem interactions and 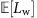 is the number of within-subsystem interactions we would expect by chance. Values of *Q*>0 (*Q*<0) mean that we observe within-subsystem interactions more (less) frequently than expected by chance. To calculate 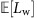, we need to choose a reference model for network structure, and here we use the Erdős–Rényi random graph^34^, as we want to contrast our results with those found for an unstructured, random network. Though *Q* is bounded by −1 and 1, only a smaller interval of values might be achievable for a given choice of a reference model, α and *C* (Methods; for example, in our case, when *α*=1/2, then *Q*∈[−1/2, 1/2], provided that *C*<1/2).

In summary, the parameterization *M*=*W* ○ *K* allows us to separate the effect on stability of a block structure (modelled by *K* through α, *C* and *Q*) from those due to the distribution of interaction strengths (modelled by *W*). Given that the case of *Q*=0 (unstructured network) has been studied intensively, and that the calculation of the stability of these matrices can be achieved analytically^20^,^32^,^33^,^35^, we use it as a reference point to determine the effect of *Q* on stability.

### Effect of modularity on stability

We want to study the effect of modularity on stability. Therefore, we contrast the value for the real part of the ‘rightmost' eigenvalue of *M*, Re(*λ*_*M*,1_), with 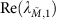, the value found for 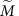, a matrix with exactly the same coefficients, but re-arranged according to a random network structure (*Q*=0). Re(*λ*_*M*,1_), is a measure of stability, as it expresses the amount of self-regulation we would need to stabilize the equilibrium^20^,^32^.

**Figure 3:**
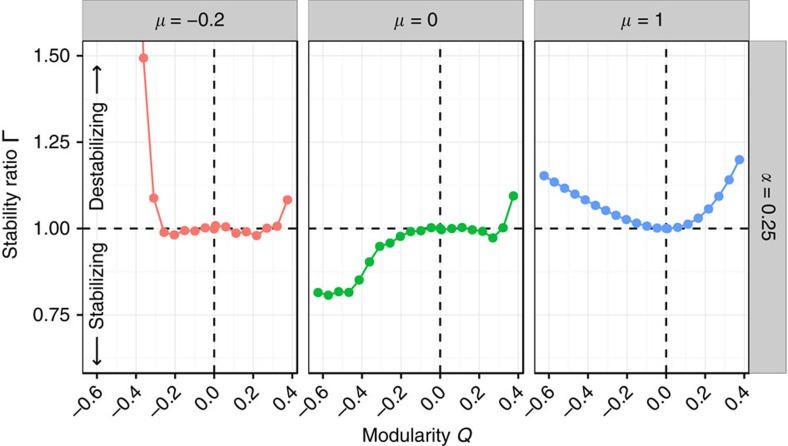
Three cases. Modularity influences stability in different ways, depending on the mean strength of interaction *μ*, the variance *σ*^2^, the correlation *ρ*, the overall connectance *C* and the size of the smaller subsystem, controlled by *α*. In this and following figures, we show the effect of the modularity (*Q*, *x* axis) on the ratio 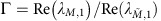 (*y* axis), measuring the stabilizing/destabilizing effect of the modularity *Q*. The ratio is obtained dividing the real part of the leading eigenvalue of the block-structured matrix, Re(*λ*_*M*,1_), by that of the corresponding unstructured matrix, 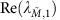. Ratios greater than one indicate destabilization, lower than one stabilization and whenever *Q*=0 (unstructured matrices) we should find Γ=1. For a given connectance (*C*=0.1 in this case), correlation (*ρ*=−3/4) and size of the smaller subsystem (*α*=1/4), we fix the variance *σ*^2^=1 and study three different cases, in which the mean *μ* is negative (red), positive (blue) or zero (green). The figure highlights that the same modularity *Q*, all other parameters being fixed, can have completely different effects on stability, depending on the value of *μ*. Each point is obtained averaging the ratio over 50 replicates of a community matrix containing 1,000 species (Methods).

Our analysis (Fig. 3; Methods) highlights that there are three main parameterizations we need to consider: (a) mean interaction strength close to zero (*μ*≈0); (b) strongly negative mean interaction strength; and (c) strongly positive mean interaction strength. Without loss of generality, we can set *σ*^2^=1, and study the effect of the modularity *Q* on the stability of the community, measured as the ratio 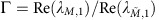, for a given choice of *α* (controlling the size of the smaller subsystem), *ρ* (correlation of interaction strengths) and *C* (overall connectance of the system). Values Γ<1 are found when imposing the block structure helps stabilizing the community (for example, in Fig. 3, for *Q*<0 and *μ*=0), while ratios Γ >1 stand for destabilizing effects (for example, any *Q*≠0 for positive mean). For an unstructured matrix (*Q*=0), the ratio is exactly 1.

**Figure 4:**
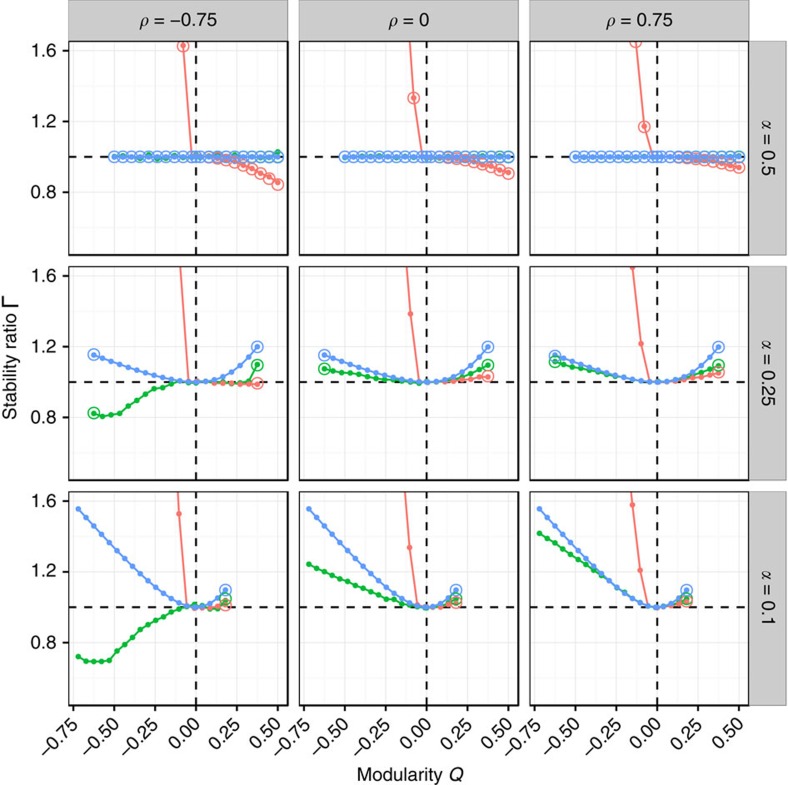
Effects of modularity on stability. For each value of *α* (determining the size of the smaller subsystem; rows) and *ρ* (expressing the correlation between interaction strengths; columns), we vary *Q* and record the ratio Γ. Given that the maximum and minimum *Q* that can be attained depend on *C* and *α* (Methods), we take 20 equally spaced points between the minimum and maximum *Q* for each configuration. We set *C*=0.2, *S*=1,000 and *σ*^2^=1, and track the case of *μ*=0 (green), *μ*=−1 (red) and *μ*=1 (blue). The dots represent numerical simulations, obtained by averaging over 50 replicates. The open circles are the corresponding analytical predictions (Methods). Because for negative means the destabilizing effect of bipartite structures is so strong that plotting it would make the other effects difficult to see, we only plot the region of Γ bounded by 1/2 and 3/2. See Supplementary Fig. 1 for the complete graph.

In Fig. 4, we show the effect of modularity on stability in a community composed of 1,000 species, when we set *C*=0.2. Take the case of two equally sized subsystems (*α*=1/2), for which we derive new results allowing us to express the ratio analytically (Methods; Supplementary Information): when *μ*≥0, we have no effect of modularity on stability; when *μ*<0, on the other hand, a bipartite structure is highly destabilizing, while a modular structure is moderately stabilizing. Both effects are stronger in the case of a negative correlation.

When the two subsystems have different sizes (*α*<1/2), the stabilizing effect of modularity found for the case of *μ*<0 is greatly diminished, and eventually also modularity can become destabilizing (especially for positive *ρ* and 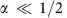). For *μ*>0, any *Q*≠0 is destabilizing, while for *μ*≈0, a modular structure is always destabilizing, and a anti-modular structure can be stabilizing, provided that *ρ* is sufficiently negative. These effects hold qualitatively for different levels of *C* (Methods; Supplementary Figs 2–3), with higher connectances leading to more marked effects. Though we cannot predict the ratio in full generality for the case of *α*<1/2, we can treat the extreme cases of a perfectly modular and perfectly bipartite structure, and this is sufficient to understand the qualitative behaviour of all cases (Methods).

The picture emerging from these results is much more nuanced and complex than what was previously hypothesized^20^,^21^,^22^: modularity can have a moderate stabilizing effect when the two subsystem have about the same size, and the mean *μ* is negative or destabilizing, when *μ*≥0 and the subsystems have different sizes. Similarly, anti-modularity is highly destabilizing for *μ*≠0, but can be stabilizing for *μ*=0.

**Figure 5:**
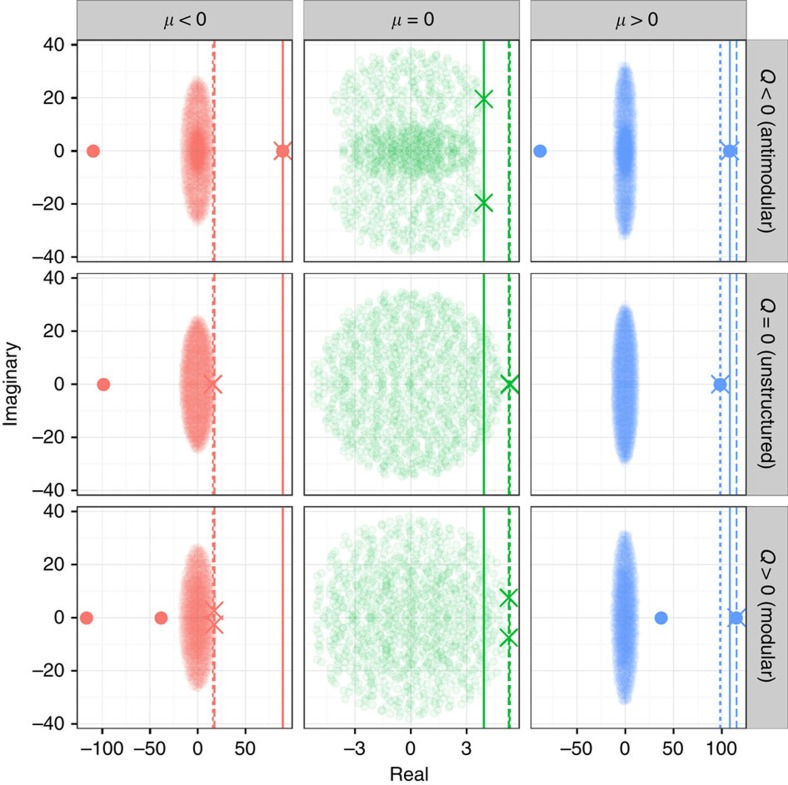
Effects of modularity on the shape of the spectrum. The spectrum of block-structured matrices is given by a ‘bulk', and—when there are two subsystems—up to two ‘outlier' eigenvalues (highlighted points). The stability of the system is determined by the rightmost eigenvalue(s) (crosses), which can either be one of the outliers (cases with *μ*>0 and when *Q*<0, *μ*<0), or the rightmost eigenvalue(s) of the bulk (cases with *μ*=0, and *μ*<0 with *Q*≤0). Large effects of *Q* on stability are found when varying *Q* changes the type of eigenvalue determining stability (*μ*<0), while, when the type of eigenvalue determining stability does not depend on *Q*, effects will be moderate. The panels show the eigenvalues of a single matrix with *S*=1,000, *C*=0.4, *α*=1/4 and *σ*^2^=1. Red: *μ*=−1/4, *ρ*=−1/4; green: *μ*=0, *ρ*=−3/4; and blue: *μ*=1/4, *ρ*=−0.5. The dashed solid line marks the position of the rightmost eigenvalue for the case of *Q*=−0.5 (top row); the dotted line for *Q*=0 (middle row) and the dashed line for *Q*=0.35 (bottom row).

The qualitative behaviour of these systems can be understood quite simply, when considering the distribution of the eigenvalues of the block-structured matrices in the complex plane. As shown in Fig. 5, when there are two subsystems the spectrum of *M* is composed of a ‘bulk' of eigenvalues, and up to two ‘outlier', real eigenvalues. When *μ*≈0, there are no outliers, and thus stability is determined in all cases by the rightmost eigenvalue(s) in the bulk. When *μ*≠0, we have only one outlier in the case of unstructured networks: if *μ*<0, then the outlier lies to the left of the bulk and thus has limited effects on stability; if *μ*>0, on the other hand, the outlier lies to the right of the bulk and therefore solely determines stability. The modular case is similar to the unstructured one, though we now have two outilers, in that both lie either to the right (*μ*>0) or the left (*μ*<0) of the bulk. In the bipartite case, however, for any *μ*≠0 the spectrum presents an outlier to the right (determining stability) and one to the left of the bulk.

These simple observations are sufficient to understand the very strong destabilizing effect of a bipartite structure when *μ*<0: in this case, the stability of the unstructured network, 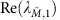, is determined by the bulk of the eigenvalues, while that of the block-structured network, Re(*λ*_*M*,1_), by the outlier to the right of the bulk (Fig. 5, red). When both Re(*λ*_*M*,1_) and 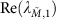 are determined by the bulk (for example, modular case with *μ*<0 or any structure with *μ*≈0), the either stabilizing or destabilizing effect is going to be moderate. Moderate effects are also observed when both Re(*λ*_*M*,1_) and 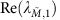 are associated with an outlier lying to the right of the bulk (*μ*>0). When both Re(*λ*_*M*,1_) and 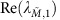 are determined by the same type (bulk, outlier) of eigenvalue, the precise stabilizing or destabilizing effect depends nonlinearly on the parameters *α*, *C*, *Q*, *μ*, *σ* and *ρ* (Methods).

To summarize, a block structure for an otherwise random ecological system can help stabilization in only two cases: (a) when the structure is modular, and *μ*<0 (though small *α* or a positive *ρ* could reverse this effect); and (b) when the structure is bipartite, *μ*≈0, and the correlation is negative. For all the other cases, the effect of a block structure ranges from neutral to highly destabilizing.

### Food-web structure

**Figure 6:**
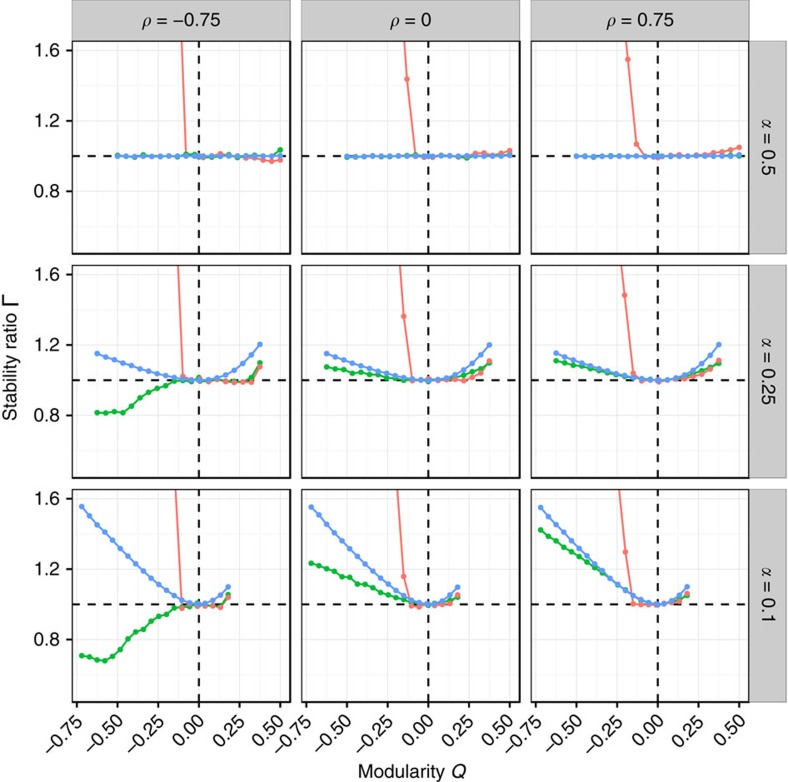
Effect of modularity on food webs. As in Fig. 4, but with the matrix *W* built such that when two species *i* and *j* interact, and species *i* is ‘smaller' than *j*, then the effect of *j* on *i*, *W*_*ij*_ is negative (on average), while that of *i* on *j* positive. This means that the food web is structured, as in a cascade model with block structure (Methods). While the results are generally similar to those in Fig. 4, food-web structure greatly decreases the stabilizing effect of modularity found when *μ*<0 and *α*≈1/2 in the random case. The same qualitative results are found when varying the connectance *C* (Supplementary Figs 4–6).

Clearly, ecological systems do not follow a random graph structure, for example, displaying a directionality in the flow of energy from producers to consumers. This directionality proved important in our previous study^36^, where we showed that when the mean of the negative effects dominates that of the positive effects, systems built according to the cascade^37^ model (in which ‘larger' species consume ‘smaller' ones) are more likely to be stable than their random counterparts. We therefore analysed matrices constructed using a variation of the cascade model, where we assign a ‘size' to each species and each species can only consume smaller species, and has a preference for those in the same subsystem (*Q*>0), or for those in the other subsystem (*Q*<0). Note that in this case, we need to set a mean for the positive effects and another one for the negative effects (Methods). Figure 6 shows that the stabilizing effect of modularity found before for the case of *μ*<0 is practically negligible, while the other results are qualitatively the same.

## Discussion

We have studied the effect of a modular or anti-modular network structure on the stability of an otherwise random ecological system. Our parameterization makes it easy to compare the effect of the network structure with that one would obtain for unstructured, random systems such as those studied in the past^20^,^33^: a ratio Γ<1 stand for stabilizing and Γ>1 for destabilizing effect of network structure.

For block-structured matrices, we showed that modularity can have a positive effect on stability only when (a) the system is composed of two subsystems of about the same size and (b) the overall mean interaction strength is negative. The stabilizing effect is stronger for negative correlations. Anti-modularity, on the other hand, is typically strongly destabilizing, besides the case in which the average interaction strength is close to 0 (a well-studied case^20^,^33^, though of limited biological realism). When the mean interaction strength is positive, both modularity and anti-modularity are destabilizing.

Through numerical simulations, we have investigated the more complex case of an interaction between modularity and food-web structure, and found that the results are qualitatively unchanged.

The picture emerging from both simulations and mathematical analysis is much more complex than previously hypothesized. Block structure can have an effect on the local asymptotic stability of the underlying system. However, unless we are in particular areas of the parameter space, the effect tends to be destabilizing. Our results stress the fact that, when discussing the relationship between network structure and local stability, we need to qualify our statements, as a given structure is not stabilizing or destabilizing *per se*, but is only so under certain specific conditions.

**Figure 7:**
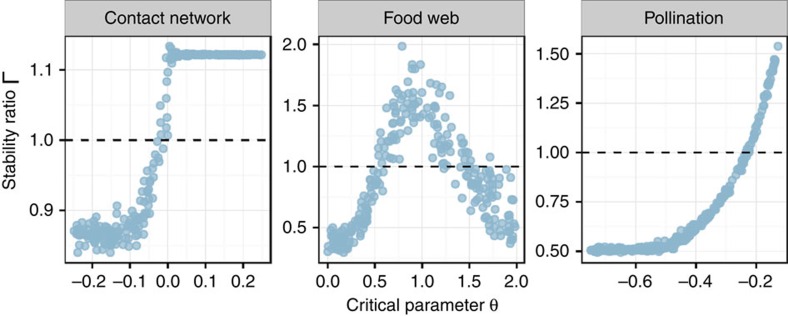
Variable effect of network structure. We show the results of numerical simulations where we parameterize empirical networks, and we study the stabilizing/destabilizing effect of network structure (Γ, *y* axis) when varying a ‘critical' parameter *θ* (*x* axis). When parameterizing a high-school social network^42^ (left), *θ* is the average of the nonzero coefficients: for negative *θ*, we observe that matrices with empirical structure are easier to stabilize than their random counterparts (Γ<1); the reverse is found for positive means. For the parameterization of the Little Rock food web^43^ (centre), *θ* expresses the relative magnitude of positive effects (effects of prey on predators) with respect to the negative ones (effects of predators on prey), so that when *θ*≈1 positive effects are about as strong as the negative ones, and network structure is destabilizing; for *θ* much lower (higher) than one, on the other hand, network structure is stabilizing. Finally, for a pollination network^44^,^45^ (right), the strength of plant–plant and pollinator–pollinator competition (*θ*) can be stabilizing (strong competition) or destabilizing (weak competition). Details on the simulations are reported in the Methods section.

Though we have illustrated this point by studying the modular structures, we believe this phenomenon to hold generally: any network structure can have different effects on stability, depending on the choice of parameters. To reinforce this message, in Fig. 7, we show three cases in which an empirical network structure makes the system more or less stable than its random counterpart, depending on the parameterization of the coefficients.

Practically, this means that the challenge of proving that biological network structure emerges because of a selective process, removing configurations yielding unfavourable dynamics^17^,^18^,^19^ is much harder than expected: network structure, without estimates of the distribution of the coefficients, cannot be used to determine the effect on dynamical properties.

## Methods

### Constructing the community matrix

*M* is the *S* × *S* community matrix, representing the population dynamics of an unknown dynamical system around a feasible equilibrium point. We consider two cases: (a) random ecological networks with block structure and (b) food webs with block structure.

For case (a), we sampled the pairs (*W*_*ij*_, *W*_*ji*_) independently from a bivariate normal distribution with means (*μ*, *μ*)^*T*^, and covariance matrix


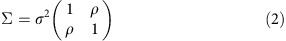


For case (b), we first assigned a ‘size' to each species (randomly sampling it from a uniform distribution between 0 and 1), and then sampled the pairs (*W*_*ij*_, *W*_*ji*_) independently from a normal bivariate distribution with means ((1+*ξ*)*μ*, (1−*ξ*)*μ*)^*T*^ (ensuring that 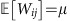, for Fig. 6, *ξ*=3), and covariance matrix Σ, whenever *i* was larger than *j*. This means that species can only consume ‘smaller' prey, such as in the cascade model. In particular, in this case we could order the rows and columns of matrix *W* so that all the positive effects would be confined to the upper-triangular part and the negative effects to the lower-triangular part.

In both cases, the matrix *M* is obtained via the Hadamard (element-by-element) product of *W* and the adjacency matrix *K*. The matrix *K* is characterized by four parameters: *S*, *α*, *C* and *Q*. *S* is the size, *α* is the proportion of species belonging to the first subsystem, *Q* is the modularity and *C* the overall connectance of *K* (density of the nonzero elements). The first *αS* species are assigned to the first subsystem, and the remaining (1−*α*)*S* species to the second subsystem. The vector 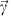 encodes the subsystem-membership of each species. Then, we set (*K*_*ij*_, *K*_*ji*_) to (1, 1) with probability *C*_w_, when *γ*_*i*_=*γ*_*j*_ and with probability *C*_b_ when *γ*_*i*_≠*γ*_*j*_. The ‘within-subsystem connectance' *C*_w_ is:


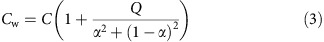


and the ‘between-subsystem connectance' *C*_b_ is:


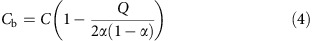


Note that, given the Erdős–Rényi reference model, the values of *Q* that are attainable depend on both *α* and *C*:


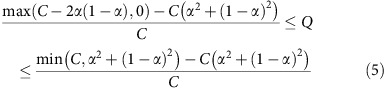


### Numerical simulations

For each *α* and *ρ*, we set *S*=1,000, *C*=0.2, *σ*=1 and *μ*=0 (green), *μ*=−1 (red) or *μ*=1 (blue), and varied *Q* from its minimum to its maximum possible value in twenty equally sized steps. For each parameter set, we produced 50 block-structured matrices *M*, and 50 unstructured matrices 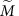, obtained by setting *Q*=0. The ratio 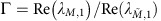 was computed by averaging over the replicates. In many cases, one can obtain the expectation for the ratio analytically (below; Supplementary Information). The simulations were repeated for both random ecological networks (Fig. 4) and cascade-based food webs (Fig. 6).

### The spectrum of block-structured matrices

For our derivations, we adopt a slightly more general notation, which includes that discussed above as a special case. We consider the matrix *M*, with *M*_*ii*_=0 and the off-diagonal coefficients independently sampled in pairs from either of two distributions:


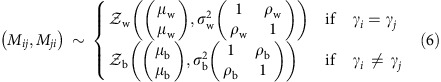


Hence, the pairs come from a certain bivariate distribution 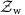, when *i* and *j* belong to the same subsystem, while from a different distribution 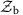, when *i* and *j* belong to different subsystems. We do not need to specify the exact form of the distributions 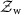 and 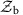, given that, as for many results in random matrix theory, our findings are consistent with the ‘universality' property^38^,^39^: once fixed the mean and covariance matrices of 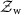 and 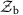, and provided that the fourth moment of each is bounded, any choice of distributions yields the same result, for *S*→∞.

When considering the case examined in the main text, where the elements *M*_*ij*_=*W*_*ij*_*K*_*ij*_, the universality property helps us in two ways. First, consider that the pairs (*M*_*ij*_, *M*_*ji*_) are zero with probability 1−*C*_*w*_ (case *γ*_*i*_=*γ*_*j*_) or probability 1−*C*_*b*_ (case *γ*_*i*_≠*γ*_*j*_), and that the nonzero pairs are sampled from a bivariate distribution (for the elements of *W*), defined by the parameters *μ*, *σ*^2^ and *ρ*. This is sufficient to calculate^32^ the relevant parameters for the distributions 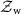 and 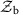:


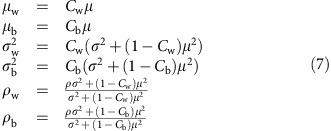


This means that the effect of the connectances is somewhat trivial: we can ‘absorb' the connectances in the parameters *μ*_w_, *μ*_b_, *σ*_w_, *σ*_b_, *ρ*_w_ and *ρ*_b_, which are our ‘effective' parameters, dictating the shape of the distribution of the eigenvalues of *M*.

The second advantage of having universal results is that we are free to choose any distribution for the pairs (*W*_*ij*_, *W*_*ji*_). In all our examples, these are sampled from a bivariate normal distribution.

### Decomposition

We want to study the limiting (that is, when *S* is large) distribution of the eigenvalues of *M* for the case of a random community (for the food webs following the cascade model, we rely exclusively on simulations). Following the approach by Allesina *et al*.^36^, we write the matrix *M* as the sum of two matrices, *M*=*A*+*B*, where *A* is a matrix with block structure whose elements are


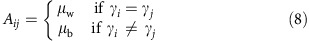


and *B* is obtained by difference: *B*=*M*−*A*. Thus, the diagonal elements of *B*_*ii*_=−*μ*_*w*_, while for the off-diagonal 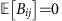, and 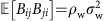 (when *γ*_*i*_=*γ*_*j*_), or 
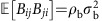
 (when *γ*_*i*_≠*γ*_*j*_).

This parameterization is very convenient, as the spectrum of matrix *B* describes the bulk of the eigenvalues of *M*, while the outlier eigenvalues of *M* are given by the nonzero eigenvalues of *A*, modified by a small correction^40^.

### The eigenvalues of *A*

The eigenvalues of *A* are easy to obtain for any choice of *S*, *α*, *μ*_w_ and *μ*_b_, with all being zero besides


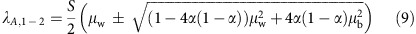


which can be both zero as well (*μ*_w_=*μ*_b_=0), both different from zero (*α*≠1/2, *μ*_w_≠*μ*_b_), or one zero and one nonzero (*μ*_w_=*μ*_b_≠0). Thus, there are going to be up to two outlier eigenvalues.

These are the approximate locations of the two outlier eigenvalues of *M* (only one outlier when *μ*_b_=*μ*_w_≠0, as found for example in the ‘unstructured' case). The exact location of the outliers depends also on *B*, as explained below.

### The eigenvalues of *B*

The spectrum of *B* has never been studied in full generality. We start by discussing the known cases, and then introduce new results that allows us to understand the qualitative behaviour of our simulations. These results can be derived by a calculation making use of the cavity method (Supplementary Information).

### Known case: *σ*_w_=*σ*_b_, *ρ*_w_=*ρ*_b_

In this case, the eigenvalues of *B* follow the ‘elliptic law'^39^, and for *S* large, are approximately uniformly distributed in an ellipse, centred at (−*μ*_w_, 0), and with horizontal semi-axis 
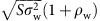
 and vertical semi-axis 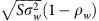.

### Known case: *σ*_b_=0 (perfectly modular)

When there are no connections between-subsystem, we have two independent subsystems. Hence, the eigenvalues of *B* are simply the union of the eigenvalues of the two squared block matrices found on the diagonal. The eigenvalues of each diagonal block follow the elliptic law, so that the distribution of the eigenvalues of *B* is a combination of two uniform ellipses, both centred at (−*μ*_w_, 0), and with horizontal semi-axes 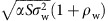 and 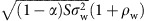, and vertical semi-axes 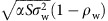 and 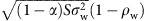, respectively.

### Known case: *ρ*_b_=*ρ*_w_=0

New results^41^ can be applied to this case, showing that the eigenvalues of *B* are contained in a circle in the complex plane, with centre (−*μ*_w_, 0) and radius


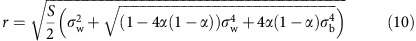


In this case, the distribution of the eigenvalues is not uniform, and Aljadeff *et al*.^41^ provide an implicit formula for the density of the limiting spectral distribution, which is consistent to that found in the Supplementary Information using a different method.

### New case: *α*=1/2 (equally sized subsystems)

When the two subsystems have the same size (*α*=1/2), we find (Supplementary Information) that the eigenvalues of *B* are approximately uniformly distributed in the ellipse in the complex plane with centre in (−*μ*_w_, 0), horizontal semi-axis


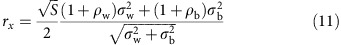


and vertical semi-axis


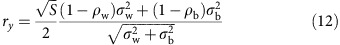


Note that this would also be the limiting distribution for the eigenvalues of the unstructured matrix 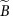 with −*μ*_w_ on the diagonal, and off-diagonal elements sampled independently in pairs from the bivariate normal distribution with means (0, 0)^*T*^, a correlation that is a weighted average of the correlations in *B*,


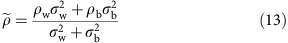


and a variance that is the arithmetic mean of the variances in *B*,


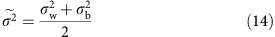


In this case, it is convenient to express these values in terms of *μ*, *σ*^2^, *ρ*, *C* and *Q*, because this makes the role of modularity in modulating the stability much clearer:


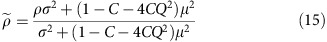



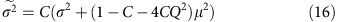


From the two equations above, it is clear that (a) for *μ*=0, modularity has no effect on the spectrum, while for *μ*≠0 the sign of *μ* does not affect the spectrum; (b) modular and bipartite structures have the same effect: the eigenvalues of *B* will be approximately the same, when we have *Q*=*q* or *Q*=−*q*; (c) the effect of modularity is going to be more marked for large *C* or |*μ*|; and (d) the radius of *B* is always lower or equal than that we would find by setting *Q*=0.

Summarizing, for *α*=1/2, the eigenvalues of *B* are contained in an ellipse whose horizontal semi-axis is always smaller or equal than that found for the corresponding unstructured matrix. This explains the stabilizing effect of a modular structure (*Q*<0) we observed for *μ*<0, as in that case the rightmost eigenvalue of *M* is the rightmost eigenvalue of *B*.

### New case: *σ*_*w*_=0 (perfectly bipartite)

When *σ*_w_=0 (that is, *C*_w_=0), the nonzero coefficients of *B* are exclusively contained in the two blocks, representing the interactions between subsystems, as in a bipartite network. Hence, we can write the matrix *B* in block form:


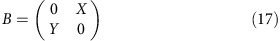


where *X* is a *αS* × (1−*α*)*S* matrix and *Y* is a (1−*α*)*S* × *αS* matrix. The two matrices on the diagonal contain all zeros, and have size *αS* × *αS* and (1−*α*)*S* × (1−*α*)*S*, respectively.

The eigenvalues of *B*^2^, are the eigenvalues of *B*, squared: if *λ*_*i*_ is an eigenvalue of *B*, then 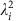 is an eigenvalue of *B*^2^. Squaring *B*, we obtain:


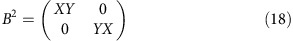


*XY* has *αS* eigenvalues, while *YX* has (1−*α*)*S* eigenvalues. The eigenvalues of *YX* are the same as those of *XY*, with the exception of (1−*α*)*S*−*αS* eigenvalues which are exactly 0 (take *v* to be an eigenvector of *XY*. Then *XYv=λv*. Let *w*=*Yv*, hence, *XYv*=*Xw*=*λv*. Finally, consider *YXw*=*Y*(*λv*)=*λYv=λw*. Thus, if *λ*≠0, it is an eigenvalue of both *XY* and *YX*). Hence, we can study the eigenvalues of *XY* (the smaller matrix) without loss of generality.

In the Supplementary Information, we show that the eigenvalues of *XY* are contained in an ellipse in the complex plane, with centre in 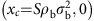, horizontal semi-axis 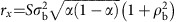 and vertical semi-axis 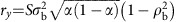 (Supplementary Fig. 7, top).

If the support of the distribution of the eigenvalues of *XY* is an ellipse in the complex plane, then the support of the eigenvalues of *B* for perfectly bipartite interaction matrices is obtained via a square-root transformation of the ellipse in the complex plane (Supplementary Fig. 7, bottom), with the addition of the point (0, 0), stemming from the extra eigenvalues of *YX*.

To find the real part of the rightmost eigenvalue of *B*, we thus need to consider the square-root transformation of the ellipse found for *XY*. The eigenvalues of *XY*, which are the squared eigenvalues of *B*, are contained in the ellipse:


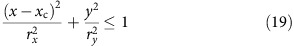


where *x* is the real part and *y* the imaginary part of the point *z*=*x*+*iy* (we can consider the case *y*>0 without loss of generality, given that the spectrum of *B* is symmetric about the real and the imaginary axis). Then, the square root of *z* has real part *a*:


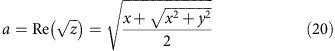


To approximate the maximum real part for the eigenvalues of *B*, we need to find the *x* that maximizes *a*. First, we can rewrite the equation for *a*, exploiting the fact that we know that all the points *z* we want to consider are on the curve describing the ellipse:


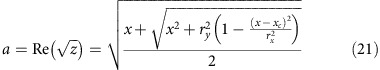


where we have substituted the value of *y*^2^ by constraining the point *z* to be on the curve describing the ellipse.

Substituting the values for *x*_*c*_, *r*_*x*_ and *r*_*y*_, we can write:


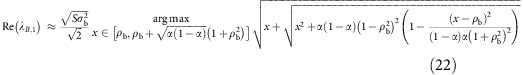


Where we maximize the function for *a* by taking values *x* in [*x*_*c*_, *x*_*c*_+*r*_*x*_], which is sufficient because of the symmetry discussed above. Maximizing, we find two cases:


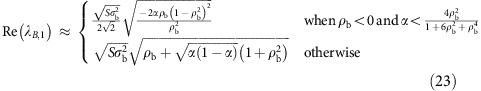


### Combining the eigenvalues of *A* and *B*

Having derived the position of the eigenvalues of *A*, and, for particular cases, the support of the distribution of those of *B*, we want to combine the results to obtain an approximation for Re(*λ*_*M*,1_), the real part of the rightmost eigenvalue of *M*=*A*+*B*.

This problem has been recently studied by O'Rourke & Renfrew^40^, who considered the following case: *B* is a large, random matrix whose eigenvalues follow the elliptic law. It is defined by its size, *S* and the distribution of the coefficients, which are independently sampled in pairs from a bivariate distribution with mean zero, unitary variance and correlation *ρ*. *A* is a matrix with low rank (that is, few nonzero eigenvalues), and nonzero eigenvalues that are sufficiently larger than those of *B*. Then (Theorem 2.4), we can order the eigenvalues of 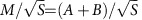 such that:


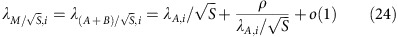


where the term *o*(1) goes to zero as *S*→∞. This means (ref. 40; Theorem 2.8) that a random matrix with a nonzero mean *μ* will have a single outlier located approximately at *μS*, exactly as found for the unstructured case^32^,^33^ studied above.

Clearly, the correction above is well suited for the unstructured case, and for the perfectly modular one (which is the combination of two unstructured cases). We also corrected in the same way the eigenvalues for matrices with *α*=1/2, reasoning that the correction would have the same form, given that the spectra of these matrices converge to those of equivalent unstructured cases. We do not have a formula for correcting the eigenvalues of bipartite matrices, but, as for the other cases, the correction is negligible when |*μ*| is large enough.

Supplementary Fig. 8 shows that our approximation is indeed excellent for all the cases considered here.

### Simulating empirical network structures

We parameterized three empirical networks, and studied the effect of network structure by measuring the ratio 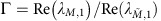, when varying a critical parameter *θ*. For simplicity, we always consider the case of matrices with zero on the diagonal.

### Contact network

We took a symmetric adjacency matrix, *A*, specifying whether two members of an high school were in contact (see ref. 42), and built the matrix *M* by sampling the coefficients *M*_*ij*_=*M*_*ji*_ from a normal distribution with mean *θ* and variance 0.0025, whenever *A*_*ij*_=*A*_*ji*_=1. We sampled *θ* independently from a uniform distribution 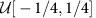 and for each *M*, we obtained 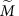 by shuffling the interactions while maintaining the pairs (so that both matrices are symmetric), following ref. 35. In Fig. 7, we show 250 realizations.

### Food web

We took the adjacency matrix *A*, specifying trophic interactions in the Little Rock lake^43^, and built *M* by sampling *M*_*ij*_ from the half-normal distribution 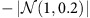, whenever *A*_*ij*_=1. These are the (negative) effects of predators on prey. For each *M*_*ij*_<0, we chose *M*_*ij*_ by multiplying −*M*_*ij*_ by a random value drawn from 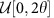. Thus, for *θ*≈1 the positive coefficients have about the same strength as the negative ones; when *θ*>1 positive effects dominate; and for *θ*<1 negative effects are stronger. Again, for each of the realizations, we built both *M* and 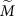, obtained by shuffling the interactions. In Fig. 7, we show results obtained by sampling *θ* 250 times from the distribution 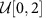.

### Pollinator network

We took the pollination network compiled by Robertson^44^,^45^, and we used the rectangular adjacency matrix to determine the position of the nonzero, mutualistic effects between plants and pollinators: *M*_*ij*_ and *M*_*ji*_ were sampled independently from the uniform distribution 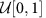, whenever plant *i* and pollinator *j* interacted. We then sampled competitive effects by sampling uniformly the coefficients 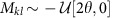 for *k* and *l* being both plants or both pollinators. Thus, *M* has a block structure, very similar to that studied here. In Fig. 7, we show the effects of the strength of competition (*θ*) on stability.

### Data availability

The data and code needed to reproduce all results presented in the article can be downloaded from https://github.com/StefanoAllesina/blockstructure

## Additional information

**How to cite this article:** Grilli, J. *et al*. Modularity and stability in ecological communities. *Nat. Commun.* 7:12031 doi: 10.1038/ncomms12031 (2016).

## Supplementary Material

Supplementary Information Supplementary Figures 1-8, Supplementary Notes and Supplementary References
