# Supplementary material for: Modularity and stability in ecological communities
Source: Nat Commun. 2016 Jun 23;7:12031. doi: 10.1038/ncomms12031 (PMC4931019; doi:10.1038/ncomms12031)
Supplement: Supplementary Information — Supplementary Figures 1-8, Supplementary Notes and Supplementary References [file ncomms12031-s1.pdf]

Supplementary Figures

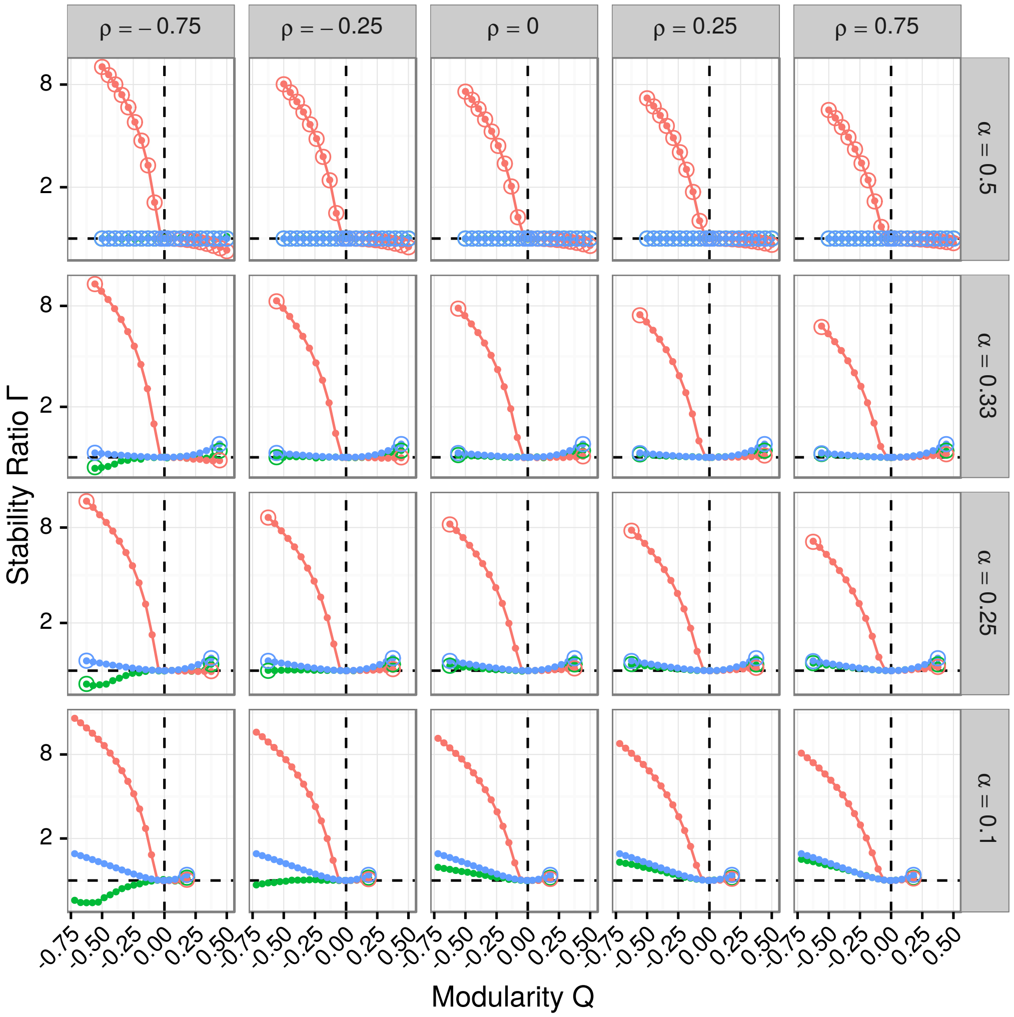

Supplementary Figure 1: Effects of modularity on stability. As Figure 4 in the main text, but showing the entire range of $\mathrm{Re}(\lambda_{\mathrm{M},1}) / \mathrm{Re}(\lambda_{\widetilde{\mathrm{M}},1})$ values. Note that the $y-$axis is the $\log_2$ of the ratio.

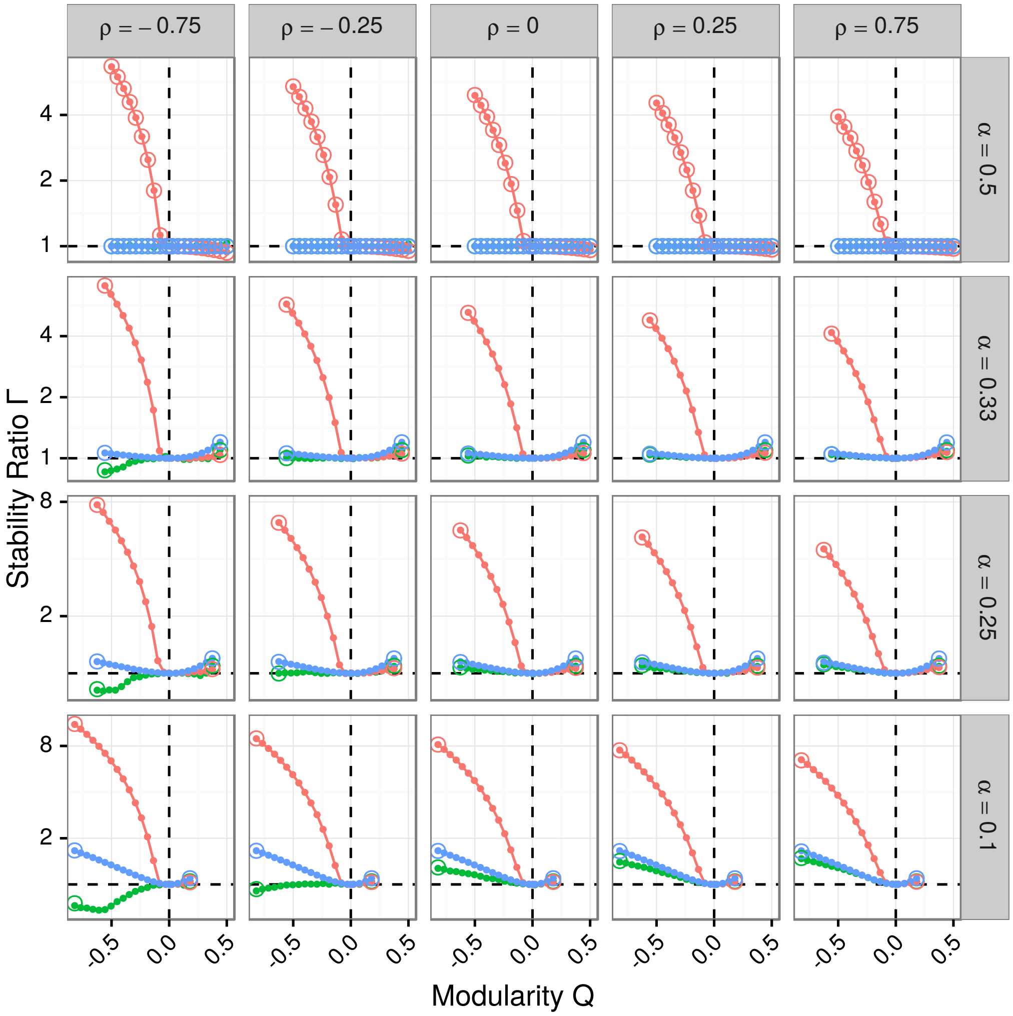

Supplementary Figure 2: Effects of modularity on stability: $C = 0.1$**.** As Supplementary Fig. 1, but for a lower connectance $C = 0.1$.

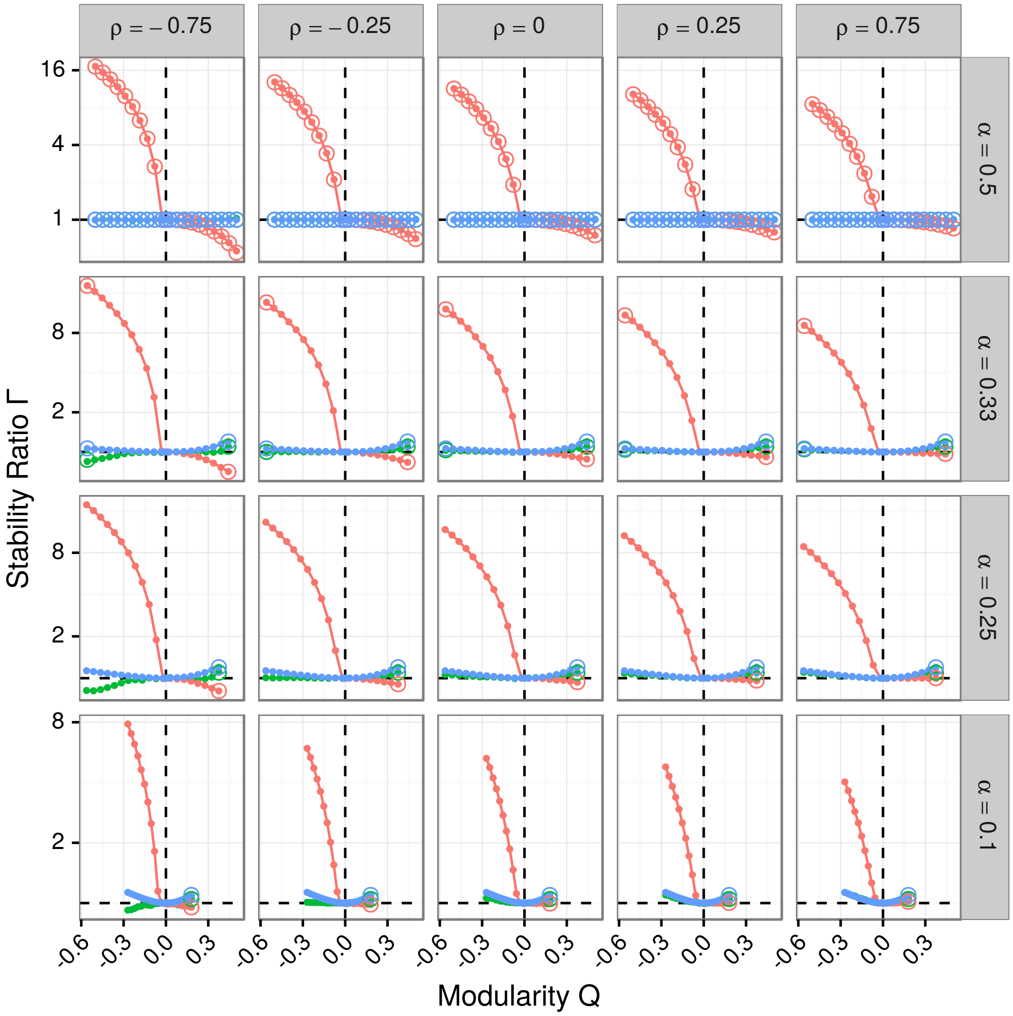

Supplementary Figure 3: Effects of modularity on stability: $C = 0.4$**.** As Supplementary Fig. 1, but for a higher connectance $C = 0.4$.

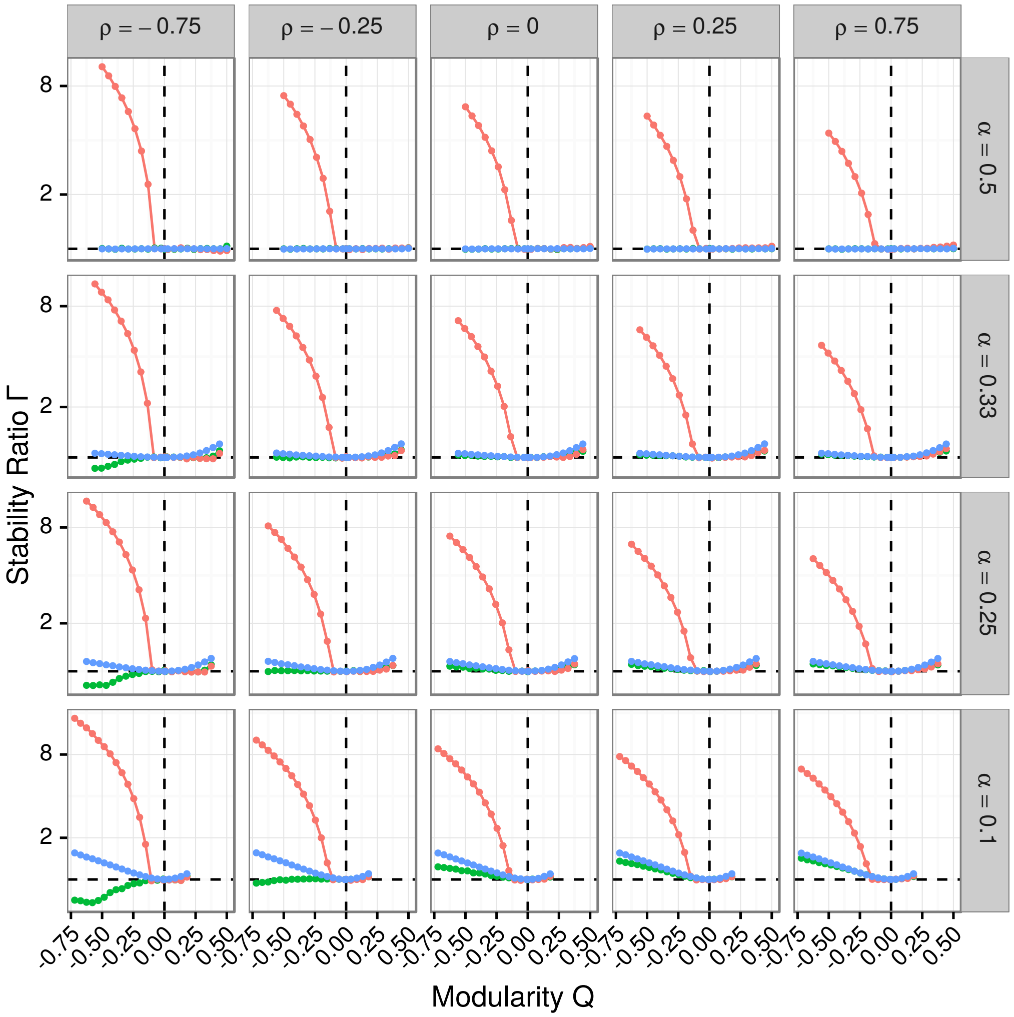

Supplementary Figure 4: Effects of modularity on food webs. As Figure 6 in the main text, but showing the entire range of $\mathrm{Re}(\lambda_{\mathrm{M},1}) \,/\, \mathrm{Re}(\lambda_{\tilde{\mathrm{M}},1})$ values. Note that the $y-$axis is the $\log_2$ of the ratio.

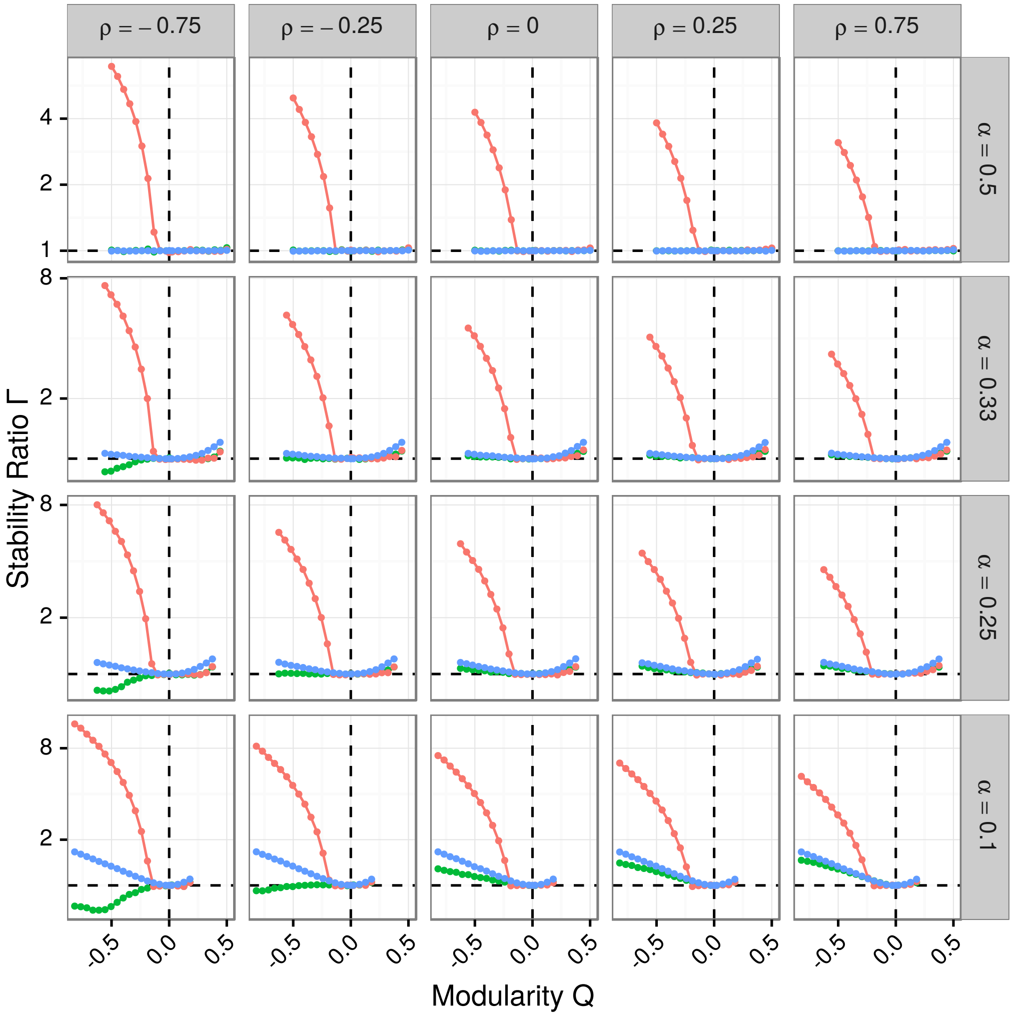

Supplementary Figure 5: Effects of modularity on food webs: $C = 0.1$**.** As Supplementary Fig. 4, but for a lower connectance $C = 0.1$.

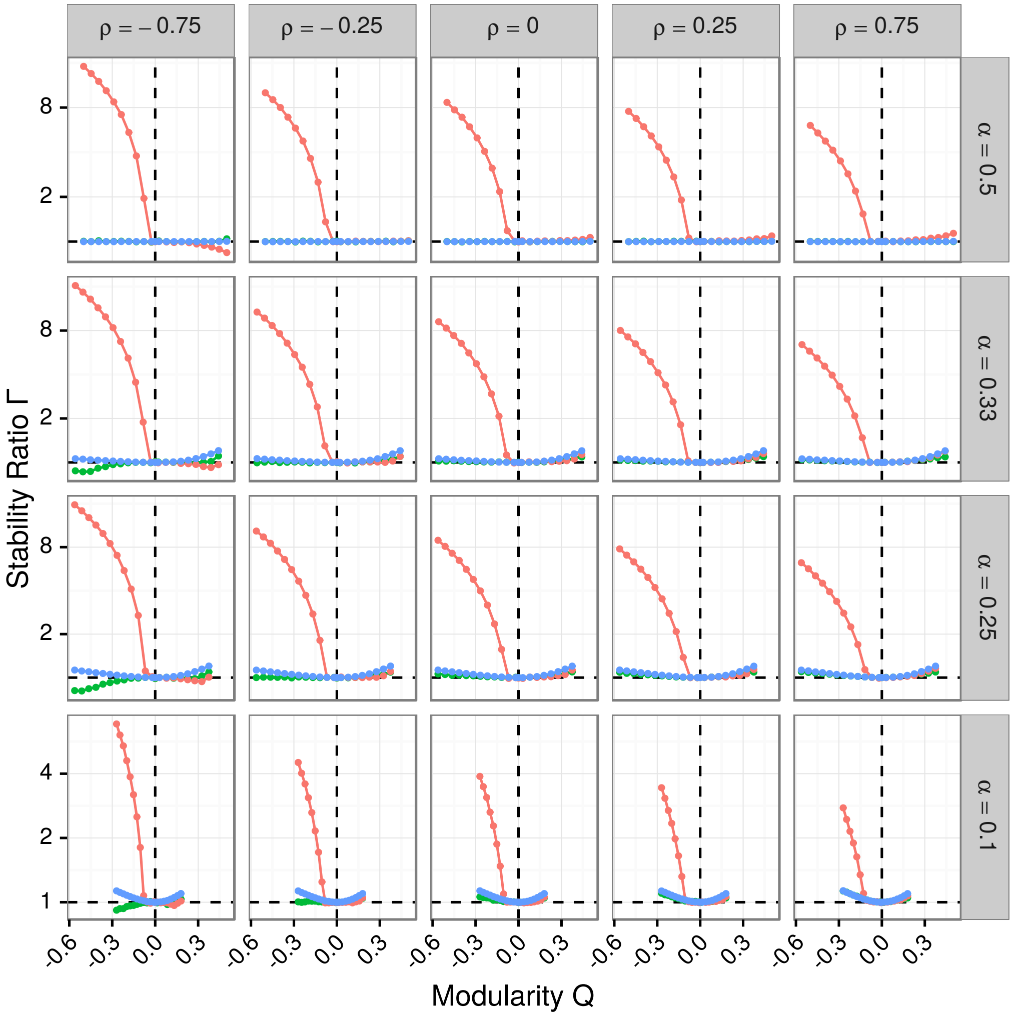

Supplementary Figure 6: Effects of modularity on food webs: $C = 0.4$**.** As Supplementary Fig. 4, but for a higher connectance $C = 0.4$.

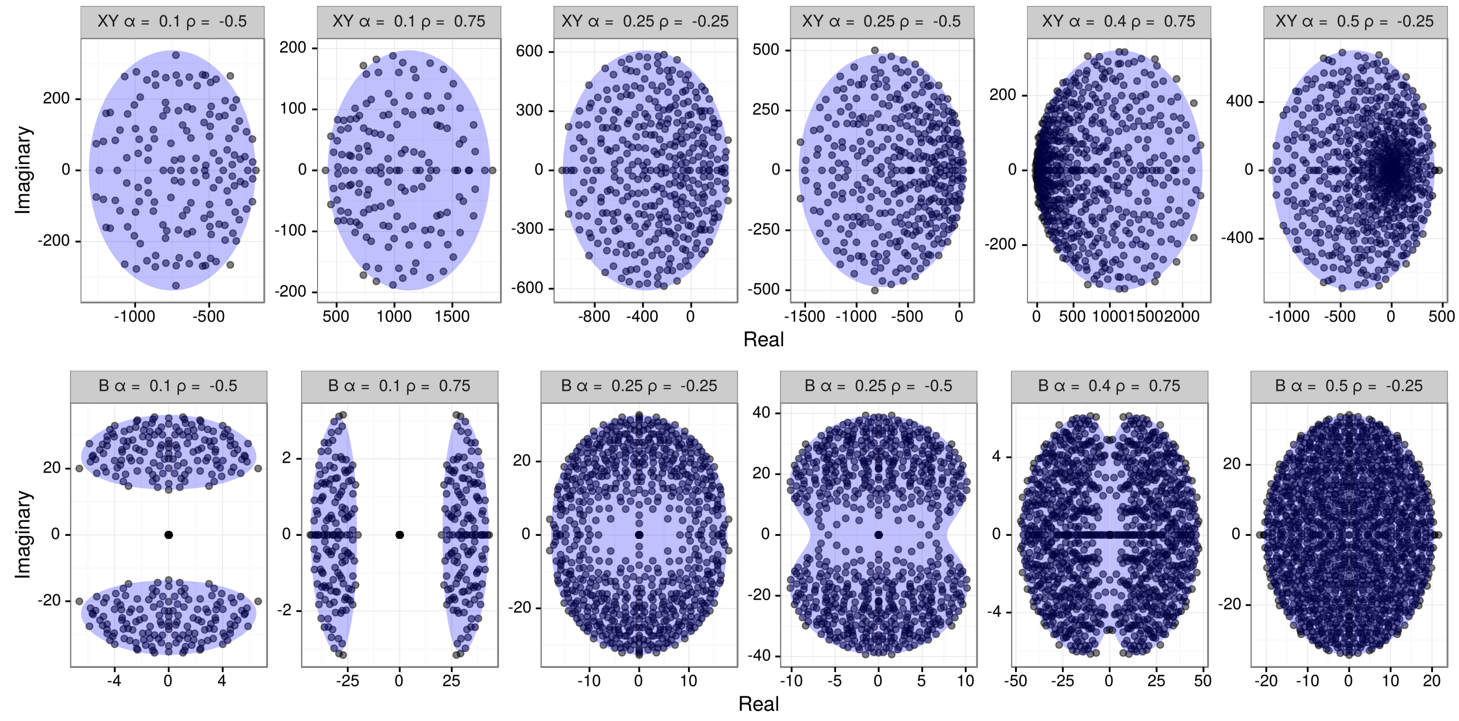

Supplementary Figure 7: The spectrum of B for bipartite matrices. When the matrix is bipartite, we can write B in block form, and the eigenvalues of B are the square roots of the eigenvalues of XY, with the addition of eigenvalues at $(0, 0)$. Top: eigenvalues of XY when we set $S = 1500$, $\sigma_b = 1$, and choose different values of ρ_b and α (panel labels). The eigenvalues (points) are approximately contained in an ellipse in the complex plane (shaded area). Bottom: this means that the eigenvalues of B (points) are in the area(s) described by the square-root transformation of the ellipse found for XY (shaded), with the addition of $(1 - \alpha)S - \alpha S$ eigenvalues at $(0, 0)$.

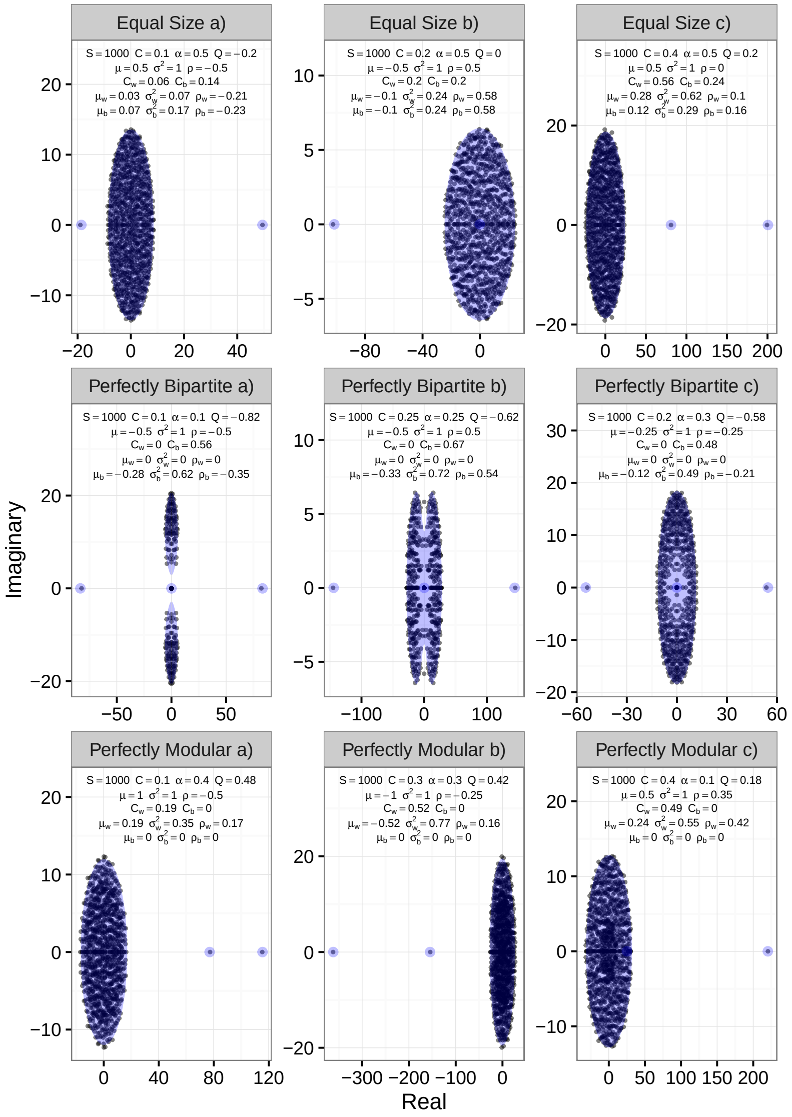

Supplementary Figure 8: Prediction of the spectrum of M. We choose a parameterization, setting the size S, connectance C, proportion of species in the first subsystem α, modularity Q, and the parameters describing the bivariate distribution, μ, $\sigma = 1$, and ρ (all reported in the panels). From these parameters, we can compute C_w and C_w, and then the "effective" parameters μ_w, μ_b, σ_w, σ_b, ρ_w, and ρ_b (also reported). From these we can derive analytically the support for the bulk of the eigenvalues, and the location of the outliers (shaded areas). Our analysis correctly predicts the actual support of the eigenvalue distribution for the case of equal-sized subsystems ($\alpha = 1/2$, top row), perfectly modular systems ($C_b = 0$, middle row), and perfectly bipartite matrices ($C_w = 0$, bottom row).

Supplementary Notes

In what follows, we present an analytical derivation of the limiting distribution for the eigenvalues of a block-structured random matrix B. The matrix as size $S \times S$, and the rows/columns (equivalently, the nodes in the networks) are assigned a group membership, encoded in the vector $\underline{\gamma}$. We consider the simple case of only two groups, in which each element $\gamma_i \in (1, 2)$. The diagonal elements of B are all equal, and set to $-\mu_w$. Since the effect of adding a constant diagonal to a matrix is simply to shift its eigenvalue distribution, we consider the simplest case of $B_{ii} = 0$, without loss of generality. The off-diagonal coefficient of B are random variables satisfying the following:

$$\begin{aligned} \mathbb{E}[B_{ij}] &= 0 \\ \mathbb{E}[(B_{ij})^2] &= \sigma_w^2 \quad \text{if } \gamma_i = \gamma_j \\ \mathbb{E}[(B_{ij})^2] &= \sigma_b^2 \quad \text{if } \gamma_i \neq \gamma_j \\ \mathbb{E}[B_{ij}B_{ji}] &= \rho_w \sigma_w^2 \text{ if } \gamma_i = \gamma_j \\ \mathbb{E}[B_{ij}B_{ji}] &= \rho_b \sigma_w^2 \text{ if } \gamma_i \neq \gamma_j \ . \end{aligned} \tag{1}$$

We first derive a closed set of equations describing the spectral distribution (more precisely, its resolvent, see Section) for arbitrary values of ρ_b, σ_b, ρ_w, and σ_w. We then investigate the cases presented in the main text, obtaining an explicit solution for the support of the spectrum of B in two special cases: when the two subsystems have the same size ($\alpha = 1/2$), and when interactions only occur between groups ($\sigma_w = 0$).

Prerequisites

Spectral distribution and Hermitian random matrices.

Given a $S \times S$ Hermitian random matrix B with eigenvalues λ_i, the spectral density is defined as

$$\varrho(\lambda) := \frac{1}{S} \sum_i \delta\left(\lambda - \lambda_i\right) \ . \tag{2}$$

In the limit of large S, this quantity converges to a limiting distribution that, with a slight abuse of notation, we can write as

$$\varrho(\lambda) := \mathbb{E}\left(\delta\left(\lambda - \lambda_i\right)\right) \ . \tag{3}$$

Our goal is to compute this average over the randomness of the matrix B. It is useful to introduce the resolvent G, defined as

$$G(z) := \frac{1}{S} \sum_i \frac{1}{\lambda_i - z} \ , \tag{4}$$

or, in terms of averages as

$$G(z) := \mathbb{E} \left(\frac{1}{S} \mathrm{Tr} \left(B - z \right)^{-1} \right) \ . \tag{5}$$

It is important to recall that, if B is an Hermitian matrix, then all of its eigenvalues are real. The resolvent is instead a complex function of the complex variable z. The resolvent and the spectral density are simply related: we have

$$G(z) = \int d\lambda \ \frac{\varrho(\lambda)}{\lambda - z} \ , \tag{6}$$

and

$$\varrho(\lambda) = -\frac{1}{\pi} \lim_{\epsilon \to 0^+} \Im \left(G(\lambda + i\epsilon) \right. \ . \tag{7}$$

Quaternions

Complex numbers are defined by introducing the purely imaginary number i, with the property $i^2 = -1$. All the algebraic properties of complex numbers descend from this fact (together with associativity, commutativity and distributive properties). For instance, the sum of two complex numbers is given by

$$(a + ib) + (x + iy) = (a + x) + (b + y)i \ , \tag{8}$$

while the product by

$$(a + ib)(x + iy) = (ax - by) + (bx + ya)i \ . \tag{9}$$

If $z = x + iy$, one can introduce the conjugate $\bar{z} = x - iy$ so that

$$|z|^2 := \bar{z}z = (x - iy)(x + iy) = x^2 + y^2 \tag{10}$$

is a real positive number. It is then easy to see that the inverse z^{-1} can be written as

$$z^{-1} = \frac{\bar{z}}{|z|^2} \ . \tag{11}$$

Complex numbers can be represented as vectors, whose entries correspond to the real and imaginary part.

Similarly, quaternions can be defined by introducing the symbols i, j, and k, with the properties

$$i^2 = j^2 = k^2 = ijk = -1 \ . \tag{12}$$

The following multiplication rules hold:

$$ij = 1 \ , \ jk = 1 \ , \ ki = 1 \ , \ ji = -1 \ , \ kj = -1 \ , \ ik = -1 \ . \tag{13}$$

The main difference between complex numbers and quaternions is that in the latter multiplication is not commutative: $ij \neq ji$. A quaternion $\mathbf{q}$ can be written as $\mathbf{q} = a + bi + cj + dk$, where a, b, c and d are real numbers. More conveniently, by using the identity $k = ij$, a quaternion can be written as $\mathbf{q} = z + wj$, where $z = a + bi$ and $w = c + di$ are complex numbers. Since i and j do not commute, it is important to stress that $wj \neq jw$. We have instead $wj = j\bar{w}$.

The sum of two quaternions is simply obtained by applying the associative property

$$(z + wj) + (\alpha + \beta j) = (z + \alpha) + (w + \beta)j \ , \tag{14}$$

while the product can be obtained using the properties of i, j and k explained above

$$\begin{aligned}(z + wj)(\alpha + \beta j) =& z\alpha + wj\beta j + z\beta j + wj\alpha \\ =& z\alpha + w\bar{\beta}j^2 + z\beta j + w\bar{\alpha}j \\ =& (z\alpha - w\bar{\beta}) + (z\beta + w\bar{\alpha})j \ .\end{aligned} \tag{15}$$

Given a quaternion $\mathbf{q} = a + bi + cj + dk = z + wj$ (where a, b, c and d are real numbers, $z = a + bi$ and $w = c + di$), one can define its conjugate

$$\bar{\mathbf{q}} = a - bi - cj - dk = \bar{z} - wj \ , \tag{16}$$

so that

$$|\mathbf{q}|^2 := \bar{\mathbf{q}}\mathbf{q} = \mathbf{q}\bar{\mathbf{q}} = |z|^2 + |w|^2 \ , \tag{17}$$

is a real positive number. As in the case of complex numbers, the inverse of a quaternion $\mathbf{q}$ is unique (i.e., $\mathbf{q}^{-1}\mathbf{q} = \mathbf{q}\mathbf{q}^{-1} = 1$) and can be written as

$$\mathbf{q}^{-1} := \frac{\bar{\mathbf{q}}}{|\mathbf{q}|^2} \ . \tag{18}$$

One can also introduce an element-by-element multiplication, defined as

$$(z + wj) \circ (\alpha + \beta j) = z\alpha + w\beta j \ . \tag{19}$$

Quaternions can be represented as matrices. In particular one can think of $\mathbf{q} = z + wj$ as the 2×2 matrix

$$\mathbf{q} = \begin{pmatrix} z & w \\ \bar{w} & \bar{z} \end{pmatrix} \ . \tag{20}$$

All the properties of sum and multiplication explained above simply follows from matrix algebra. When representing quaternions as matrices, the element-by-element product $\circ$ corresponds to the Hadamard product. The numbers i, j and k, when expressed in matrix notation, turn out to be (proportional to) the three Pauli matrices.

Spectral distribution and non-Hermitian random matrices

In the case of non-Hermitian random matrices, the eigenvalues need not to be real, and in general lay in the complex plane. Writing the eigenvalues as $\lambda = x + yi$, the spectral density can be defined as

$$\varrho(x, y) = \frac{1}{S} \sum_{i=1}^{S} \delta\left(x - \Re\left(\lambda_i\right)\right) \delta\left(y - \Im\left(\lambda_i\right)\right) \ . \tag{21}$$

As we saw above, for Hermitian matrices the eigenvalues are real, while the resolvent is a complex function. In the non-Hermitian case, the eigenvalues are complex, and the resolvent is a quaternion function:

$$\mathcal{G}(\mathbf{q}) = \frac{1}{S} \sum_{i} \left(\lambda_i - \mathbf{q}\right)^{-1} \ , \tag{22}$$

where q is a quaternion.

The resolvent can be expressed in terms of the spectral density

$$\mathcal{G}(\mathbf{q}) = \int dxdy \;\varrho(x,y)\,(x+iy-\mathbf{q})^{-1} \;. \tag{23}$$

On the other hand, the spectral density can be easily obtained from the resolvent

$$\varrho(x,y) = -\frac{1}{\pi}\lim_{\epsilon\to 0^+} \Re\left(\frac{\partial}{\partial\bar{\lambda}}\mathcal{G}(\lambda+\epsilon j)\right)\Big|_{\lambda=x+iy} \;, \tag{24}$$

where we used the notation

$$\frac{\partial}{\partial\bar{\lambda}} := \frac{1}{2}\left(\frac{\partial}{\partial x} + i\frac{\partial}{\partial y}\right) \;. \tag{25}$$

Cavity method for non-Hermitian random matrices

The cavity method was introduced in statistical physics, to solve models with tree-like interactions [1]. In the context of random matrices [2, 3], the cavity method is exact for tree-like matrices and is a good approximation in the case of sparse matrices. We are, on the other hand, interested in large, densely connected matrices. Interestingly, for this case of large S and high-connectivity, the cavity solution is expected to be exact, and it reduces to a simple set of equations for the resolvent.

A full derivation of the cavity equations can be found in the articles by Rogers and collaborators [2, 3]. Here we use a slightly different notation of the cavity equations based on quaternions, rather than Pauli matrices. The mapping between quaternions and Pauli matrices allows to recover the original results.

We introduce the resolvent matrix

$$\mathbf{G} = (\mathbf{B}-\mathbf{q})^{-1} \;, \tag{26}$$

where $\mathbf{q}$ is the quaternion

$$\mathbf{q} = \lambda + \epsilon j = \begin{pmatrix} \lambda & i\epsilon \\ i\epsilon & \bar{\lambda} \end{pmatrix} \;, \tag{27}$$

while $\mathbf{B}$ is a $2S \times 2S$ block-matrix with structure

$$\mathbf{B}_{ij} = \begin{pmatrix} B_{ij} & 0 \\ 0 & B_{ji} \end{pmatrix} . \tag{28}$$

Then, the resolvent becomes

$$\mathcal{G}(\mathbf{q}) = \frac{1}{S} \sum_i \mathbf{G}_{ii}(\mathbf{q}) . \tag{29}$$

Assuming that B_{ij} has a tree structure (i.e., there are no closed loops), one can apply the cavity method [3], obtaining the following equations for the diagonal entries of $\mathbf{G}$

$$\mathbf{G}_{ii} = -\left(\mathbf{q} + \sum_{j \neq i} \mathbf{B}_{ij} \mathbf{G}_{jj}^{(i)} \mathbf{B}_{ji} \right)^{-1} \tag{30}$$

where $\mathbf{G}^{(i)}$ is the resolvent of the matrix obtained by removing row and column i from B. This can be expressed as

$$\mathbf{G}_{ii}^{(k)} = -\left(\mathbf{q} + \sum_{j \neq i,k} \mathbf{B}_{ij} \mathbf{G}_{jj}^{(i)} \mathbf{B}_{ji} \right)^{-1} . \tag{31}$$

Solving iteratively these equations, and using equation 24, one can evaluate the spectral density.

Cavity equations for block-structured matrices

When S is large, several important simplifications of equations 30 and 31 occur:

1. At the leading order in S, the right side of equation 30 is identical for all i in the same group, so we may write $\mathbf{G}_{jj} = \mathbf{G}_{\gamma_j}$.
2. Since S is large, removing a single node i does not change the leading order behavior of the system, so we can use $\mathbf{G}_{jj}^{(i)} = \mathbf{G}_{jj} = \mathbf{G}_{\gamma_j}$. This implies that equation 30 becomes a closed equation and we do not need equation 31 anymore.

3. We can apply the law of large numbers to approximate the sum in the right-hand side of equation 30, obtaining therefore, at the leading order in S

$$\sum_{j\neq i,k} \mathbf{B}_{ij}\mathbf{G}_{jj}^{(i)}\mathbf{B}_{ji} \approx \mathbb{E}\left(\sum_j \mathbf{B}_{ij}\mathbf{G}_{\gamma_j}\mathbf{B}_{ji}\right) \tag{32}$$

Using equation 28 and the matrix representation of quaternions, we obtain

$$\mathbf{B}_{ij}\mathbf{G}_{\gamma_j}\mathbf{B}_{ji} = \begin{pmatrix} B_{ij} & 0 \\ 0 & B_{ji} \end{pmatrix}\begin{pmatrix} r_{\gamma_j} & \beta_{\gamma_j} \\ \bar{\beta}_{\gamma_j} & \bar{r}_{\gamma_j} \end{pmatrix}\begin{pmatrix} B_{ji} & 0 \\ 0 & B_{ij} \end{pmatrix} = \begin{pmatrix} B_{ij}B_{ji}r_{\gamma_j} & B_{ij}^2\beta_{\gamma_j} \\ B_{ji}^2\bar{\beta}_{\gamma_j} & B_{ij}B_{ji}\bar{r}_{\gamma_j} \end{pmatrix} , \tag{33}$$

where we used the notation $\mathbf{G}_\gamma = r_\gamma + \beta_\gamma j$. In the case of two blocks, we have that, for an arbitrary vector with components z_{γ_j},

$$\mathbb{E}\left(\sum_j B_{ij}^2 z_{\gamma_j}\right) = \sum_j \mathbb{E}\left(B_{ij}^2\right) z_{\gamma_j} = \sum_j \left(\delta_{\gamma_i,\gamma_j}\sigma_w^2 z_{\gamma_j} + (1-\delta_{\gamma_i,\gamma_j})\sigma_b^2 z_{\gamma_j}\right) \tag{34}$$

Since we are considering only two blocks, γ_i assumes only two values. When considering $\gamma_i = 1$, we have

$$\mathbb{E}\left(\sum_j B_{ij}^2 z_{\gamma_j}\right) = S\alpha\sigma_w^2 z_1 + S(1-\alpha)\sigma_b^2 z_2 \qquad \text{if } \gamma_i = 1 \ , \tag{35}$$

where α is the fraction of elements belonging to the block 1. For $\gamma_i = 2$, we obtain instead

$$\mathbb{E}\left(\sum_j B_{ij}^2 z_{\gamma_j}\right) = S(1-\alpha)\sigma_w^2 z_2 + S\alpha\sigma_b^2 z_1 \qquad \text{if } \gamma_i = 2 \ . \tag{36}$$

Similarly, by using the expectation value of $\mathbb{E}\left(B_{ij}B_{ji}\right)$, we find

$$\mathbb{E}\left(\sum_j B_{ij}B_{ji} z_{\gamma_j}\right) = S\alpha\rho_w\sigma_w^2 z_1 + S(1-\alpha)\rho_b\sigma_b^2 z_2 \qquad \text{if } \gamma_i = 1 \ , \tag{37}$$

and

$$\mathbb{E}\left(\sum_j B_{ij}B_{ji}z_{\gamma_j}\right) = S(1-\alpha)\rho_w\sigma_w^2 z_2 + S\alpha\rho\sigma_b^2 z_1 \qquad \text{if } \gamma_i = 2 \ . \tag{38}$$

Substituting in equation 33, we obtain

$$\begin{aligned}\mathbb{E}\left(\sum_j \mathbf{B}_{ij}\mathbf{G}_{\gamma_j}\mathbf{B}_{ji}\right) &= \begin{pmatrix} \mathbb{E}\left(\sum_j B_{ij}B_{ji}r_{\gamma_j}\right) & \mathbb{E}\left(\sum_j B_{ij}^2\beta_{\gamma_j}\right) \\ \mathbb{E}\left(\sum_j B_{ji}^2\bar{\beta}_{\gamma_j}\right) & \mathbb{E}\left(\sum_j B_{ij}B_{ji}\bar{r}_{\gamma_j}\right) \end{pmatrix} \\ &= S\alpha\sigma_w^2\begin{pmatrix} \rho_w r_1 & \beta_1 \\ \bar{\beta}_1 & \rho_w\bar{r}_1 \end{pmatrix} + S(1-\alpha)\sigma_b^2\begin{pmatrix} \rho_b r_2 & \beta_2 \\ \bar{\beta}_2 & \rho_b\bar{r}_2 \end{pmatrix} \qquad \text{if } \gamma_i = 1 \ . \end{aligned} \tag{39}$$

By introducing

$$\mathbf{\Sigma}_w = S\sigma_w^2(\rho_w + j) \quad \text{and} \quad \mathbf{\Sigma}_b = S\sigma_b^2(\rho_b + j) \ , \tag{40}$$

the previous expression can be written more compactly

$$\mathbb{E}\left(\sum_j \mathbf{B}_{ij}\mathbf{G}_{\gamma_j}\mathbf{B}_{ji}\right) = \alpha\mathbf{\Sigma}_w \circ \mathbf{G}_1 + (1-\alpha)\mathbf{\Sigma}_b \circ \mathbf{G}_2 \qquad \text{if } \gamma_i = 1 \ . \tag{41}$$

A similar expression can be obtained in the other case

$$\mathbb{E}\left(\sum_j \mathbf{B}_{ij}\mathbf{G}_{\gamma_j}\mathbf{B}_{ji}\right) = (1-\alpha)\mathbf{\Sigma}_w \circ \mathbf{G}_2 + \alpha\mathbf{\Sigma}_b \circ \mathbf{G}_1 \qquad \text{if } \gamma_i = 2 \ , \tag{42}$$

where $\circ$ is the element by element product.

Armed with the calculations and the simplifications explained above, equation 30 can be reduced to

$$\mathbf{G}_1 = -\left(\mathbf{q} + \alpha\mathbf{\Sigma}_w \circ \mathbf{G}_1 + (1-\alpha)\mathbf{\Sigma}_b \circ \mathbf{G}_2\right)^{-1} \ , \tag{43}$$

and

$$\mathbf{G}_2 = -\left(\mathbf{q} + \alpha\mathbf{\Sigma}_b \circ \mathbf{G}_1 + (1-\alpha)\mathbf{\Sigma}_w \circ \mathbf{G}_2\right)^{-1} \ . \tag{44}$$

The resolvent is then given by $\mathcal{G} = \alpha\mathbf{G}_1 + (1-\alpha)\mathbf{G}_2$, and the spectral density can be obtained from equation 24. As considered above we can use the general form

$$\mathbf{G}_1 = r_1 + \beta_1 j \ , \ \mathbf{G}_2 = r_2 + \beta_2 j \ , \tag{45}$$

where r_1, r_2, β_1, β_2 are, at least in principle, complex numbers. In practice, we show that the support of the spectral distribution is defined by the existence of a solution with real and positive values of β_1 and β_2. The existence of such a solution determines the support of the spectral distribution, thereby bounding the maximum real part of the eigenvalues of B.

Explicit solutions

In the Section above, we considered the general case of B with a block-structure with two groups and arbitrary α, ρ_b, σ_b, ρ_w, and σ_w. In this Section, we provide an explicit solution for the support of the spectrum of B in two particular cases.

New case 1, $\alpha = 1/2$.

If $\alpha = 1/2$, the right sides of equation 43 and 44 become equal, so that

$$\mathbf{G}_1 = \mathbf{G}_2 =: \mathbf{G} = r + \beta j \ , \tag{46}$$

where $\mathbf{G}$ is a solution of

$$\mathbf{G} = -\left(\mathbf{q} + \frac{\mathbf{\Sigma}_w + \mathbf{\Sigma}_b}{2} \circ \mathbf{G}\right)^{-1} \ . \tag{47}$$

It is natural to introduce

$$\tilde{\mathbf{\Sigma}} := \frac{\mathbf{\Sigma}_w + \mathbf{\Sigma}_b}{2} = S\tilde{\sigma}^2\left(\tilde{\rho} + j\right) \ , \tag{48}$$

where

$$\tilde{\sigma}^2 = \frac{\sigma_w^2 + \sigma_b^2}{2} \ , \quad \tilde{\rho} = \frac{\rho_w\sigma_w^2 + \rho_b\sigma_b^2}{\sigma_w^2 + \sigma_b^2} \ . \tag{49}$$

Multiplying both sides of equation 50 by its right-hand side, we obtain

$$\mathbf{G}\left(-\mathbf{q} - \tilde{\mathbf{\Sigma}} \circ \mathbf{G}\right) = 1 \ , \tag{50}$$

Using equation 46 and 27, and setting $\epsilon = 0$, we obtain

$$(r + \beta j)\left(-\lambda - S\tilde{\sigma}^2(\tilde{\rho}r + \beta j)\right) = 0 \ , \tag{51}$$

that, after separating the part multiplied by j from the other part, reduces to two equations

$$r\left(-rS\tilde{\rho}\tilde{\sigma}^2 - \lambda\right) + S|\beta|^2\tilde{\sigma}^2 = 1 \ , \tag{52}$$

and

$$\beta\left(-rS\tilde{\rho}\tilde{\sigma}^2 - \lambda - S\bar{r}\tilde{\sigma}^2\right) = 0 \ . \tag{53}$$

The spectral density is given by

$$\varrho(\lambda) = -\frac{1}{\pi}\mathrm{Re}\frac{\partial r}{\partial \bar{\lambda}} \ . \tag{54}$$

If $\beta = 0$, equation 52 reduces to

$$r\left(-rS\tilde{\rho}\tilde{\sigma}^2 - \lambda\right) = 1 \ . \tag{55}$$

Taking the derivative of both sides respect to $\bar{\lambda}$,

$$\frac{\partial r}{\partial \bar{\lambda}}\left(-2rS\tilde{\rho}\tilde{\sigma}^2 - \lambda\right) = 0 \ , \tag{56}$$

which implies that $\partial r/\partial\bar{\lambda} = 0$. The solution $\beta = 0$ corresponds to values of λ outside the support of the spectral distribution.

If $\beta \neq 0$, equation 53 is solved by

$$r = \frac{1}{S\tilde{\sigma}^2}\left(-\frac{x}{1+\tilde{\rho}} + \frac{iy}{1-\tilde{\rho}}\right) \ , \tag{57}$$

and then, from equation 52, we obtain

$$|\beta|^2 = \frac{1}{S\tilde{\sigma}^2}\left(1 - \frac{x^2}{S(1+\tilde{\rho})^2\tilde{\sigma}^2} - \frac{y^2}{S(1-\tilde{\rho})^2\tilde{\sigma}^2}\right) \ . \tag{58}$$

Since $|\beta|^2$ is a positive real value, a solution $\beta \neq 0$ exists only if the right side of the previous equation is positive, i.e., when

$$\frac{x^2}{S(1+\tilde{\rho})^2\tilde{\sigma}^2} + \frac{y^2}{S(1-\tilde{\rho})^2\tilde{\sigma}^2} < 1 \ , \tag{59}$$

which corresponds to an ellipse in the complex plane. Outside of these region, the only solution is $\beta = 0$ and the spectral density is null. Inside this region the spectral density can be obtained from equation 57

$$\varrho\lambda = -\frac{1}{\pi}\mathrm{Re}\frac{\partial r}{\partial\bar{\lambda}} = \frac{2}{\pi S}\frac{1}{(1-\tilde{\rho}^2)} \ . \tag{60}$$

In the case $\alpha = 1/2$, the density is therefore constant inside an ellipse with semi-axes

$$r_x = \frac{\sqrt{S}}{2}\tilde{\sigma}(1+\tilde{\rho}) \ , \quad r_y = \frac{\sqrt{S}}{2}\tilde{\sigma}(1-\tilde{\rho}) \ . \tag{61}$$

New case 2, $\sigma_w = 0$.

If $\sigma_w = 0$, equations 43 and 44 reduce to

$$\mathbf{G}_1 = -\left(\mathbf{q} + (1-\alpha)\mathbf{\Sigma}_b \circ \mathbf{G}_2\right)^{-1} \ , \tag{62}$$

and

$$\mathbf{G}_2 = -\left(\mathbf{q} + \alpha\mathbf{\Sigma}_b \circ \mathbf{G}_1\right)^{-1} \ , \tag{63}$$

which can be written as a single equation for $\mathbf{G}_1$

$$\mathbf{G}_1 = \left(-\mathbf{q} + (1-\alpha)\mathbf{\Sigma}_b \circ \left(\mathbf{q} + \alpha\mathbf{\Sigma}_b \circ \mathbf{G}_1\right)^{-1}\right)^{-1} \ . \tag{64}$$

We obtain therefore

$$\mathbf{G}_1^{-1} = -\mathbf{q} + (1-\alpha)\mathbf{\Sigma}_b \circ \left(\mathbf{q} + \alpha\mathbf{\Sigma}_b \circ \mathbf{G}_1\right)^{-1} \ , \tag{65}$$

and, by introducing

$$\mathbf{G}_1 = r + \beta j \ , \tag{66}$$

we get

$$\begin{aligned}\frac{\bar{r} - \beta j}{|r|^2 + |\beta|^2} &= -\lambda + (1-\alpha)S\sigma_b^2(\rho_b + j) \circ \frac{\bar{\lambda} + \alpha S\sigma_b^2\rho_b\bar{r} - \alpha S\sigma_b^2\beta j}{|\lambda + \alpha S\sigma_b^2\rho_b r|^2 - |\alpha S\sigma_b^2\beta|^2} = \\ &= -\lambda + (1-\alpha)S\sigma_b^2\frac{\rho_b\bar{\lambda} + \alpha S\sigma_b^2\rho_b^2\bar{r} - \alpha S\sigma_b^2\beta j}{|\lambda + \alpha S\sigma_b^2\rho_b r|^2 - |\alpha S\sigma_b^2\beta|^2} \ ,\end{aligned} \tag{67}$$

where we already considered the limit $\epsilon \to 0$. We obtain two independent equations

$$\beta\left(\frac{1}{|r|^2+|\beta|^2}-\alpha(1-\alpha)\frac{(S\sigma_b^2)^2}{|\lambda+\alpha S\sigma_b^2\rho_b r|^2+|\alpha S\sigma_b^2\beta|^2}\right)=0\ , \tag{68}$$

and

$$\frac{\bar{r}}{|r|^2+|\beta|^2}=-\lambda+(1-\alpha)\frac{S\sigma_b^2\rho_b}{|\lambda+\alpha S\sigma_b^2\rho_b r|^2+|\alpha S\sigma_b^2\beta|^2}(\bar{\lambda}+\alpha S\sigma_b^2\rho_b\bar{r})\ . \tag{69}$$

As in the case of $\alpha = 1/2$, $\beta = 0$ is always solution. By substituting it in equation 69, we find that the solution corresponds to a null spectral density. We can therefore obtain the support of the spectral distribution by studying the values of λ for which a solution $\beta \neq 0$ exists. In order to find the spectral density we consider

$$\frac{1}{|r|^2+|\beta|^2}=\alpha(1-\alpha)\frac{(S\sigma_b^2)^2}{|\lambda+\alpha S\sigma_b^2\rho_b r|^2+|\alpha S\sigma_b^2\beta|^2}\ , \tag{70}$$

together with equation 69. This system of equations can be solved, obtaining

$$\begin{aligned}|\beta|^2=&-\frac{1}{2\alpha^2\left(\rho^2-1\right)^2\left(x^2+y^2\right)}\Big(\left(\rho^2-1\right)^2\left(2\alpha^2\left(\rho^2+1\right)-\alpha\left(\rho^2+3\right)+1\right)+\\&2\left(\rho^2+1\right)x^4+2x^2\left((2\alpha-1)\rho\left(\rho^2-1\right)+2\left(\rho^2+1\right)y^2\right)+\\&+\left(\alpha\left(\rho^4-1\right)-\rho^2+2\rho x^2-2\rho y^2+1\right)\sqrt{(1-2\alpha)^2\left(\rho^2-1\right)^2+4x^4+8x^2y^2+4y^4}+\\&+2\left(\rho^2+1\right)y^4-2(2\alpha-1)\rho\left(\rho^2-1\right)y^2\Big)\ ,\end{aligned} \tag{71}$$

where we set $S\sigma_b^2 = 1$ and $\rho_b = \rho$, in order to simplify the notation. The general formula can be obtained by substituting in the latter equation x and and y with $x/(\sqrt{S}\sigma_b)$ and $y/(\sqrt{S}\sigma_b)$ respectively. Since the argument of the square root appearing in equation 71 is always positive, this solution is a feasible if and only if it is positive. The line $|\beta|^2 = 0$ defines the boundary of the support. By imposing $|\beta|^2 = 0$, after few simplifications, one obtains

$$\begin{aligned}0=&(\rho^2-1)^2x^4+(\rho^2-1)^2x^4+2x^2\rho(\rho^2-1)^2+2y^2\rho(\rho^2-1)^2+\\&+2x^2y^2(\rho^4+6\rho^2+1)+(\rho^2-1)^2\left((\alpha-1)\rho^2+\alpha\right)\left(\alpha\rho^2+(\alpha-1)\right)\ .\end{aligned} \tag{72}$$

In principle, at this stage we should obtain $\mathbf{G}_2$ from equation 63, and then the support of the spectral density as the union of the regions where a solution $\beta \neq 0$ exists. On the other hand, we can immediately realize that $\mathbf{G}_2$ can be obtained from $\mathbf{G}_1$ under the exchange $\alpha \rightarrow (1-\alpha)$. Since equation 72 is invariant under the exchange $\alpha \rightarrow (1-\alpha)$, it already defines the boundary of the spectral density.

Equation 72 can be simplified by considering the change of variable

$$(a+ib) = (x+iy)^2 \ . \tag{73}$$

When expressed in terms of a and b, equation 72 reduces to

$$(a-\rho)^2\left(1-\rho^2\right)^2 + b^2\left(1+\rho^2\right)^2 = (1-\alpha)\alpha\left(1+\rho^2\right)^2\left(1-\rho^2\right)^2 \ , \tag{74}$$

which is the equation of an ellipse. The support of the spectral density can therefore be described as the square root transformation of an ellipse in the complex plane. After re-introducing the dependence on $S\sigma_b$, by using the transformations $a \rightarrow a/(S\sigma_b^2)$ and $b \rightarrow b/(S\sigma_b^2)$, we obtain the equation of the ellipse:

$$(a-S\sigma_b^2\rho_b)^2\left(1-\rho^2\right)^2 + b^2 S\left(1+\rho^2\right)^2 = (S\sigma_b^2)^2(1-\alpha)\alpha\left(1+\rho^2\right)^2\left(1-\rho^2\right)^2 \ , \tag{75}$$

which describes an ellipse centered in $(S\sigma_b^2\rho_b, 0)$, with horizontal semi-axis $S\sigma_b^2\sqrt{(1-\alpha)\alpha}\,(1+\rho^2)$, and vertical semi-axis $S\sigma_b^2\sqrt{(1-\alpha)\alpha}\,(1-\rho^2)$.

Supplementary References

[1] Mézard, M., Parisi, G., and Virasoro, M.-A. *Spin glass theory and beyond.* World Scientific Publishing, (1990).

[2] Rogers, T., Castillo, I. P., Kühn, R., and Takeda, K. Cavity approach to the spectral density of sparse symmetric random matrices. *Physical Review E* **78**(3), 031116 (2008).

[3] Rogers, T. and Castillo, I. P. Cavity approach to the spectral density of non-Hermitian sparse matrices. *Physical Review E* **79**(1), 012101 (2009).
